# Association between Genetic Polymorphisms and Risk of Kidney Posttransplant Diabetes Mellitus: A Systematic Review and Meta-Analysis

**DOI:** 10.1155/2022/7140024

**Published:** 2022-03-08

**Authors:** Shan Xu, Zhenwei Jiang, Nan Hu

**Affiliations:** ^1^Department of Pharmacy, Changzhou No. 2 People's Hospital, The Affiliated Hospital of Nanjing Medical University, Changzhou, China; ^2^Department of Pharmacy, The Third Affiliated Hospital of Soochow University, The First People's Hospital of Changzhou, Changzhou, China

## Abstract

**Objectives:**

The purpose of this study was to clarify the role of genetic factors on posttransplant diabetes mellitus (PTDM) risk.

**Methods:**

Relevant publications were systematically retrieved from PubMed, EMBASE, and the Cochrane Library up to December 2020. Data from eligible case-control and cohort studies were extracted for qualitative and quantitative analyses. Odds ratios (ORs) and 95% confidence intervals (CIs) were used to estimate the association between gene polymorphisms and PTDM in the quantitative meta-analysis.

**Results:**

A total of 43 eligible articles were identified, and 16 studies on 9 DNA variants from 8 genes were included in the meta-analysis. *TCF7L2* rs7903146 was significantly associated with PTDM risk in 5 genetic models (OR (95% CI): allelic: 1.59 (1.17–2.16), *P*=0.003; dominant recessive: 1.62 (1.14, 2.31), *P*=0.007; recessive: 1.87 (1.18, 2.94), *P*=0.007; homozygote: 2.21 (1.23, 3.94), *P*=0.008; and heterozygote 1.50 (1.08, 2.10), *P*=0.017). *KCNQ1* rs2237892 was significantly correlated with PTDM risk in 3 genetic models (allelic: 0.68 (0.58, 0.81), *P* < 0.001; dominant: 0.6 (049, 0.74), *P* < 0.001; and heterozygote: 0.61 (0.48, 0.76), *P* < 0.001). *KCNJ11* rs5219 was significantly linked with PTDM in the recessive genetic model (1.59 (1.01, 2.50), *P*=0.047). No significant correlations of PTDM with *TCF7L2* rs12255372, *SLC30A8* rs13266634, *PPARγ* rs1801282, *CDKN2A/B* rs10811661, *HHEX* rs1111875, and *IGF2BP2* rs4402960 polymorphisms were found.

**Conclusions:**

The gene polymorphisms of *TCF7L2* rs7903146, *KCNQ1* rs2237892, and *KCNJ11* rs5219 may predispose kidney transplant recipients to PTDM. Large sample size studies on diverse ethnic populations were warranted to confirm our findings.

## 1. Introduction

PTDM is a common serious complication after kidney transplantation, which is often associated with increased risk of graft failure, cardiovascular disease, and mortality [1]. Approximately 5.5% to 60.2% of kidney transplant patients develop PTDM in the first year after surgery [2]. A large retrospective study involving 11,659 kidney recipients from the United States Renal Data System (USRDS) demonstrated that the cumulative incidence of PTDM was 9.1%, 16%, and 24% at 3 months, 12 months, and 36 months, respectively [3]. Its etiopathogenesis is multifactorial, and transplant-related risk factors for PTDM include immunosuppressants, ethnicity, age, sex, body mass index, genetic factors, hepatitis C and cytomegalovirus infections, and family history of diabetes [2]. Immunosuppressive drugs consisting of corticosteroids and calcineurin inhibitors are important risk factors of PTDM, contributing to the development of hyperglycemia and diabetes [4]. Tacrolimus (TAC) and cyclosporin (CsA) are two major calcineurin inhibitors required after transplantation to prevent acute or chronic graft rejections [1]. The mechanisms underlying the diabetogenic effect of immunosuppressive regimen include enhancing insulin resistance, reducing insulin secretion, and direct toxic effects on pancreatic *β*-cells [4]. It has also been suggested that glucocorticoid-induced hyperglycemia is partially reversible through avoidance or early withdrawal of the drugs [5].

More evidence suggests that genetic risk factors play a significant role in the development of PTDM. Many genes associated with diabetes mellitus (DM) have also been correlated with PTDM risk. Gene mutations such as single nucleotide polymorphisms (SNPs) are the most common type of genetic variation. SNPs of *TCF7L2* rs7903146, *TCF7L2* rs12255372, *KCNQ1* rs2237892, *KCNJ11* rs5219, *SLC30A8* rs13266634, *PPARγ* rs1801282, *CDKN2A/B* rs10811661, *HHEX* rs1111875, and *IGF2BP2* rs4402960 have recently been detected and shown to affect PTDM occurrence. Among them, *TCF7L2* rs7903146 had an established strong effect across different populations and is the most common susceptible gene for PTDM [6–12]. One previous meta-analysis assessed the potential association between *TCF7L2* rs7903146 polymorphism and PTDM [13]. However, there was a lack of systematic review on the correlation between other genes polymorphisms and PTDM. The meta-analysis by Benson et al. evaluated the allelic distribution of 18 gene polymorphisms in PTDM development [14]. In this study, we included several updated articles and comprehensively examined the association of nine SNPs from eight genes including TCF7L2, KCNQ1, KCNJ11, SLC30A8, PPAR*γ*, CDKN2A/B, HHEX, and IGF2BP2 with PTDM risk in all allelic and genotype models. Moreover, we reviewed the literature on genetic SNP markers susceptible to PTDM, which might help predict the risk of PTDM and facilitate the early prevention of this disease.

## 2. Materials and Methods

### 2.1. Literature Search

According to the Preferred Reporting Items for Systematic Reviews and Meta-Analyses (PRISMA) guideline (see Supplementary Materials), we systematically searched PubMed, EMBASE, and the Cochrane Library for studies published up to December 2020.

### 2.2. Eligibility Criteria

The inclusion criteria included (1) kidney transplant recipients diagnosed with new-onset diabetes after transplantation (NODAT) or PTDM according to ADA or WHO guideline, (2) original studies examining the relationship between the gene polymorphism and NODAT or PTDM in patients after kidney transplantation, (3) study type: cohort or case-control studies, and (4) language restricted to English.

### 2.3. Search Strategy

When searching for possible eligible studies in the PubMed, EMBASE, and Cochrane Library databases, we used the mesh term of “kidney transplantation,” “polymorphism, genetic,” “posttransplant diabetes mellitus,” and “new-onset diabetes mellitus after transplantation,” as well as relevant keywords.

### 2.4. Data Extraction and Quality Assessment

The selection and inclusion of studies were performed in two stages by two independent reviewers, which included the analysis of titles/abstracts followed by the full texts. Disagreements were resolved by a third reviewer. Data retrieved from the eligible studies consisted of main demographical and clinical variables, including names of authors, publication year, study design, country, ethnicity, mean age, mean BMI, female percentage, genetic risk factors for PTDM, genotyping method and genotypes, diagnosis of PTDM, immunosuppressive therapy, time of PTDM diagnosis after transplantation, and age at transplant. We selected SNPs that showed significant associations with PTDM in allelic and/or genotype models from individual studies. The outcome was the evaluation of the impact of SNPs on the development of PTDM. Excel spreadsheet was used for the collection of extracted data. The methodological quality of included studies was evaluated by NOS. The base information was shown in Supplementary Table 1, and data used for all analyses were shown in Supplementary Table 2.

### 2.5. Statistical Analysis

Crude ORs with their 95% CIs were estimated and used to assess the strength of correlations of PTDM with TCF7L2 (rs7903146) C/T, TCF7L2 (rs12255372) G/T, SLC30A8 (rs13266634) C/T, KCNQ1 (rs2237892) C/T, PPAR*γ* (rs1801282) C/G, CDKN2A/B (rs10811661) C/T, HHEX (rs1111875) C/T, IGF2BP2 (rs4402960) G/T, and KCNJ11 (rs5219) C/T polymorphism. The pooled OR was calculated for allelic effect of C/T, G/T, or C/G; dominant model of CC/CT + TT, GG/GT + TT, or CC/CG + GG; recessive model of TT/CC + CT, TT/GG + GT, or GG/CC + GC; homozygote model of CC/TT, GG/TT, or GG/CC; and heterozygote model of CT/CC, GT/GG, or GC/GG. The significance of the pooled OR was determined by the *Z*-test (*P* ≤ 0.05).

Cochran's Q statistic was used to assess the heterogeneity among studies (*P* < 0.10 indicated evidence of heterogeneity; https://doi.org/10.1136/bmj.327.7414.557). When significant heterogeneity (*P* < 0.10) was achieved, the random-effects model was used to combine the effect sizes of the included studies; otherwise, the fixed-effects model was adopted [15]. In addition, sensitivity analyses were performed to identify the effects of individual studies on pooled results and test the reliability of the estimates. All statistical analyses were performed using the STATA SE 14.0 software (StataCorp, College Station, Texas, USA).

## 3. Results

### 3.1. Study Selection and Characteristics of Included Studies

**Figure 1:**
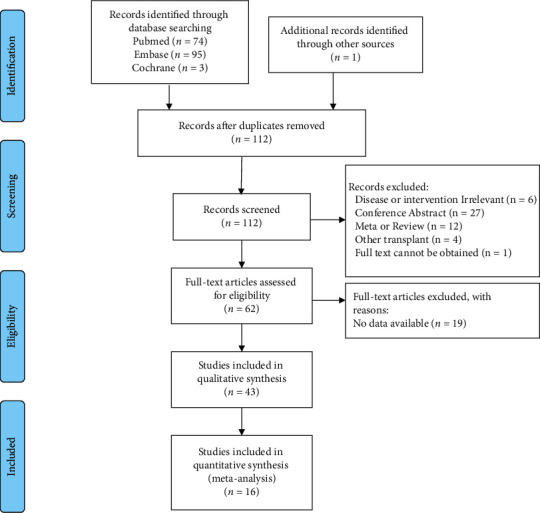
Flowchart of the search process of our study.

A total of 173 relevant publications were identified through searching the databases and other resources. After initial screening, duplicated documents; conference abstracts; reviews; publications on unrelated diseases, transplants, and interventions; and articles without full text were removed. The remaining 62 publications were assessed carefully; then 19 articles were excluded due to insufficient data. Finally, 43 eligible studies were included for the qualitative analysis. Among them, the data from 16 studies were retrieved for the quantitative meta-analysis. The study screening flow chart was shown in Figure 1. The characteristics of the selected studies for qualitative analysis were summarized in Table 1, which covered a total of 2,849 PTDM patients and 9,816 non-PTDM patients after undergoing renal transplantation. The overall incidence of PTDM varied from 8% to 42% at 3 months after transplantation and from 17% to 46% at 12 months. There were 40 retrospective or prospective cohort studies, and the rest were all retrospective case-control studies. Except for the study by Kao [16], most patients received a TAC-based treatment regimen, mainly combined with CsA, MMF, or steroid. Generally, the diagnosis of PTDM was in accordance with ADA or WHO guidelines. The mean age of patients at transplantation was 35.4 to 60 years old. The follow-up time after transplantation ranged from 1 to 36 months. The quantitative meta-analysis consisted of 16 studies involving 1,455 PTDM patients and 4,483 non-PTDM patients.

### 3.2. Quality Assessment

The quality assessment of included studies using NOS was shown in Table 2, with the maximum of 9 points representing the least risk of bias. Overall, the methodological quality scores were 9 for 24 studies, 8 for 13 studies, 7 for 4 studies, and 6 for the other 2 studies, suggesting moderate to low risk of bias. The majority of the studies in the meta-analysis had a very low bias. Among them, 12 studies were assigned 9 points; 3 studies received 8 points; and only 1 study got 7 points.

### 3.3. Meta-Analysis of the Association between Nine Genetic Polymorphisms and PTDM Risk after Renal Transplantation

**Figure 2:**
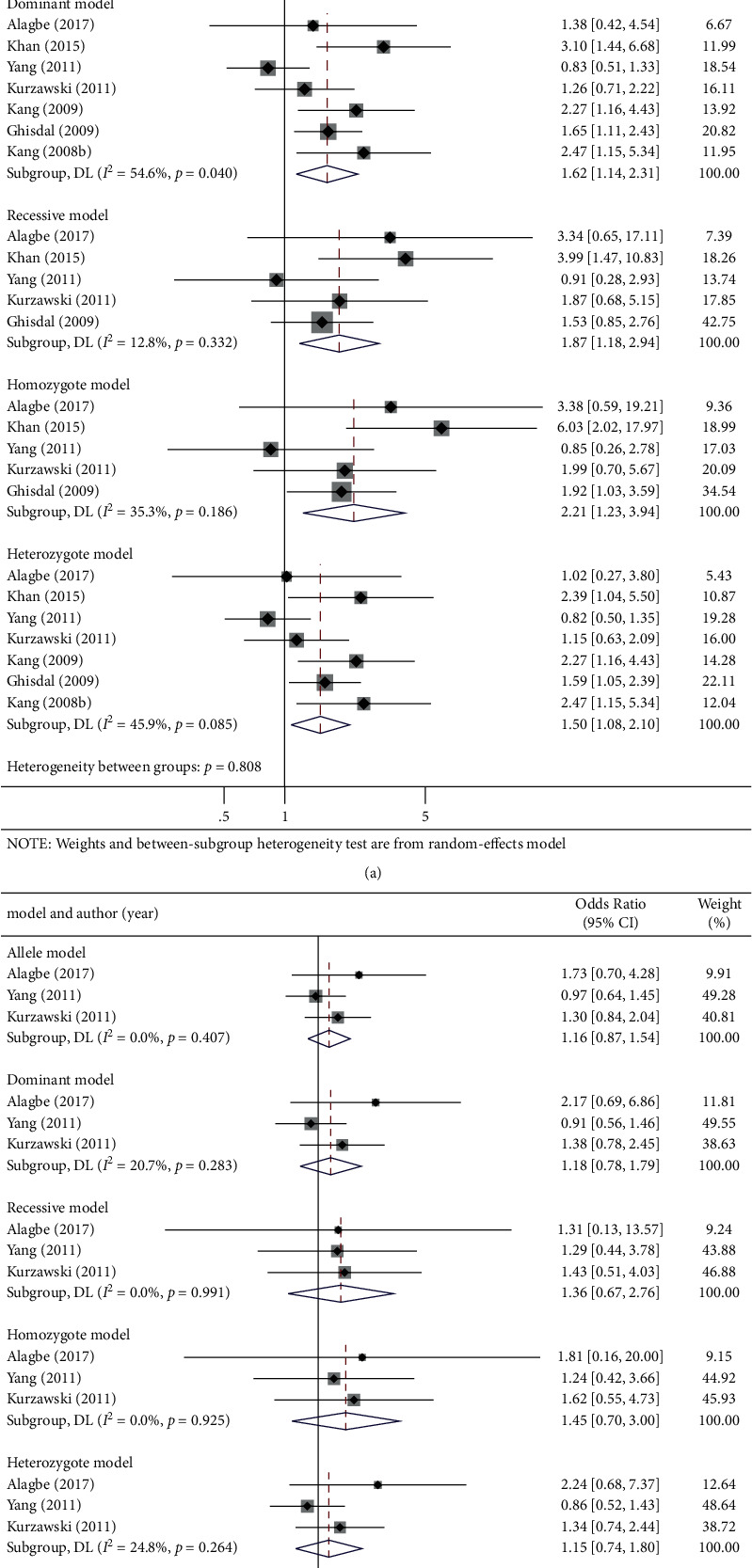
Forest plots of (a) TCF7L2 (rs7903146) C/T and (b) TCF7L2 (rs12255372) G/T polymorphism and PTDM risk in five genetic models: allele, dominant, recessive, homozygote, and heterozygote genetic model.

In this meta-analysis, the *TCF7L2* rs7903146 polymorphism was found to be significantly associated with the risk of PTDM in five genetic models (OR (95% CI): allelic: 1.59 (1.17–2.16), *P*=0.003; dominant recessive: 1.62 (1.14, 2.31), *P*=0.007; recessive: 1.87 (1.18, 2.94), *P*=0.007; homozygote: 2.21 (1.23, 3.94), *P*=0.008; and heterozygote 1.50 (1.08, 2.10), *P*=0.017; Figure 2(a) and Table 3).

The pooled analysis did not observe the susceptibility of *TCF7L2* rs12255372 polymorphism to PTDM in five genetic models (OR (95% CI): allelic: 0.16 (0.87, 1.54), *P*=0.314; dominant recessive: 1.18 (0.78, 1.79), *P*=0.424; recessive: 1.36 (0.67, 2.76), *P*=0.401; homozygote: 1.45 (0.70, 3.00), *P*=0.317; and heterozygote 1.15 (0.74, 1.81), *P*=0.529; Figure 2(b) and Table 3).

**Figure 3:**
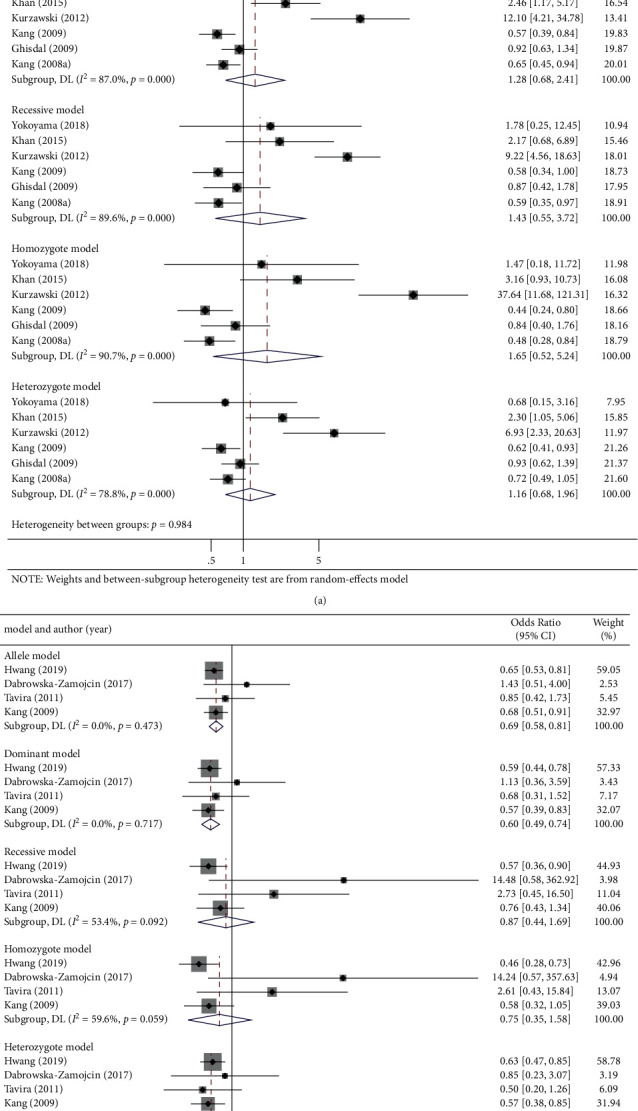
Forest plots of (a) SLC30A8 (rs13266634) C/T, (b) KCNQ1 (rs2237892) C/T, polymorphism and PTDM risk in five genetic models: allele, dominant, recessive, homozygote, and heterozygote genetic model.

*SLC30A8* rs13266634 polymorphism was not found to be significantly correlated with PTDM in five genetic models (OR (95% CI): allelic: 1.28 (0.70, 2.32), *P*=0.421; dominant: 1.29 (0.68, 2.44), *P*=0.442; recessive: 1.43 (0.55, 3.72), *P*=0.467; homozygote: 1.66 (0.52, 5.30), *P*=0.396; and heterozygote 1.16 (0.68, 1.97), *P*=0.593; Figure 3(a) and Table 3).

There was a linkage between *KCNQ1* rs2237892 polymorphism with PTDM in three genetic models (OR (95% CI): allelic: 0.68 (0.58, 0.81), *P* < 0.001; dominant: 0.6 (049, 0.74), *P* < 0.001; and heterozygote: 0.61 (0.48, 0.76), *P* < 0.001), but the association was not observed in other two genetic models (OR (95% CI): recessive: 0.87 (0.44, 1.69), *P*=0.672, and homozygote: 0.75 (0.35, 1.58), *P*=0.444; Figure 3(b) and Table 3).

**Figure 4:**
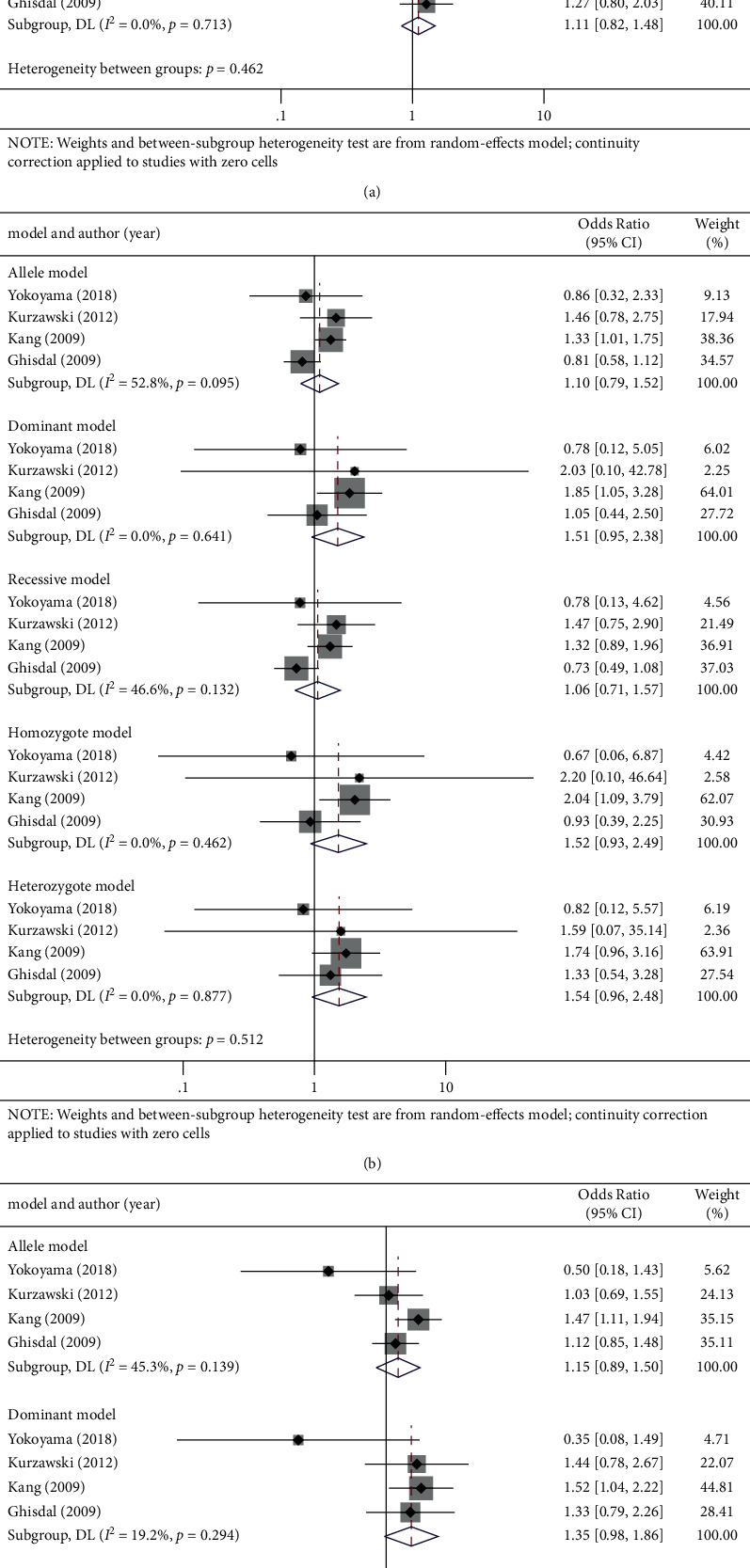
Forest plots of (a) PPAR*γ* (rs1801282) C/G, (b) CDKN2A/B (rs10811661) C/T, and (c) HHEX (rs1111875) C/T polymorphism and PTDM risk in five genetic models: allele, dominant, recessive, homozygote, and heterozygote genetic model.

Regarding PPAR*γ* rs1801282 polymorphism, no significant correlation was found in all five genetic models (OR (95% CI): allelic: 0.98 (0.75, 1.28), *P*=0.885; dominant: 1.04 (0.78, 1.40), *P*=0.772; recessive: 0.44 (0.12, 1.60), *P*=0.213; homozygote: 0.44 (0.12, 1.61), *P*=0.217; and heterozygote: 1.11 (0.82, 1.48), *P*=0.505; Figure 4(a) and Table 3).

*CDKN2A/B* rs10811661 polymorphism was also not shown to be related with PTDM risk in all five genetic models (OR (95% CI): allelic: 1.10 (0.79, 1.52), *P*=0.588; dominant: 1.51 (0.95, 2.38), *P*=0.079; recessive: 1.06 (0.71, 1.57), *P*=0.778; homozygote: 1.52 (0.93, 2.49), *P*=0.092; and heterozygote: 1.54 (0.96, 2.48), *P*=0.075; Figure 4(b) and Table 3).

With regard to *HHEX* rs1111875 polymorphism, no significant correlation with PTDM risk was demonstrated in all five genetic models (OR (95% CI): allelic: 1.15 (0.89, 1.50), *P*=0.283; dominant: 1.35 (0.98, 1.86), *P*=0.067; recessive: 1.09 (0.65, 1.83), *P*=0.735; homozygote: 1.30 (0.74, 2.30), *P*=0.357; and heterozygote: 1.35 (1.00, 1.84), *P*=0.051; Figure 4(c) and Table 3).

**Figure 5:**
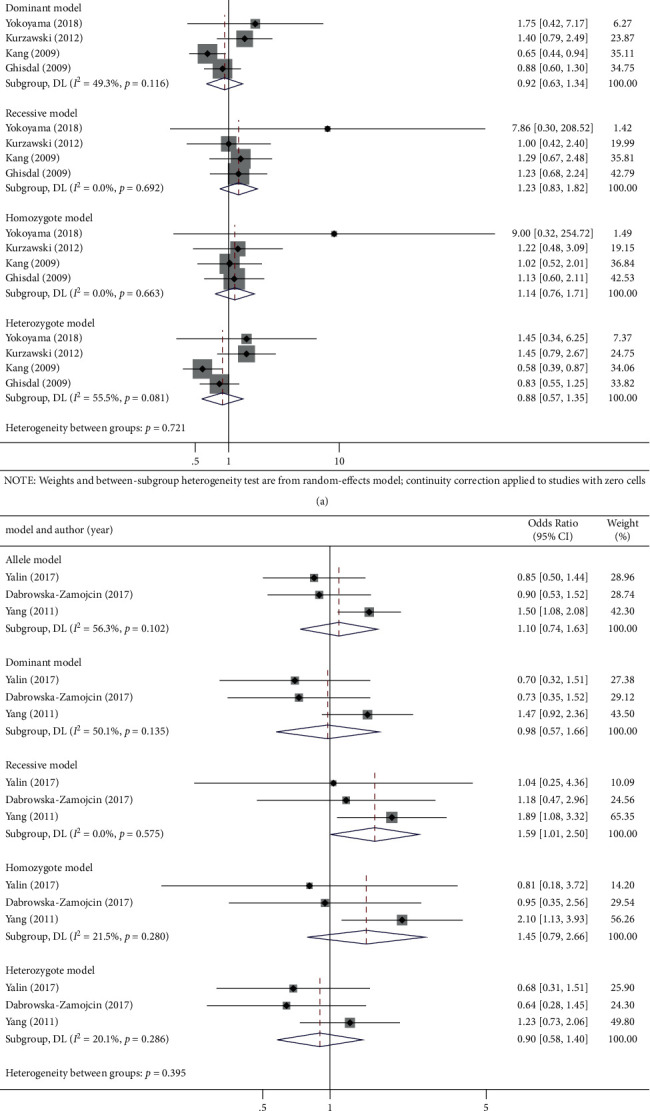
Forest plots of (a) IGF2BP2 (rs4402960) G/T and (b) KCNJ11 (rs5219) C/T polymorphism and PTDM risk in five genetic models: allele, dominant, recessive, homozygote and heterozygote genetic model.

Similarly, the *IGF2BP2* rs4402960 polymorphism was not significantly associated with PTDM in all five genetic models (OR (95% CI): allelic: 0.97 (0.78, 1.21), *P*=0.801; dominant: 0.92 (0.63, 1.34), *P*=0.670; recessive: 0.23 (0.83, 1.82), *P*=0.292; homozygote: 1.14 (0.76, 1.71), *P*=0.532; and heterozygote: 0.88 (0.57, 1.36), *P*=0.559; Figure 5(a) and Table 3).

In addition, the overall analysis revealed that *KCNJ11* rs5219 polymorphism was significantly associated with PTDM risk in the recessive genetic model (OR (95% CI): 1.59 (1.01, 2.50), *P*=0.047), though no association was found in the other genetic models (OR (95% CI): allelic: 1.10 (0.74, 1.63), *P*=0.651; dominant: 0.98 (0.57, 1.66), *P*=0.929; heterozygote: 0.90 (0.58, 1.40), *P*=0.641; and homozygote: 1.45 (0.79, 2.66), *P*=0.228; Figure 5(b) and Table 3).

### 3.4. Sensitivity Analysis

For meta-analyses on the association of three gene polymorphisms including *TCF7L2* rs7903146, *SLC30A8* rs13266634, and *PPARγ* rs1801282 with PTDM risk, the sensitivity analysis results showed that in all five genetic models, the reestimated ORs were all similar to the overall effects when excluding any individual study and assessing the remaining ones (Supplementary Figures 1–3).

## 4. Discussion

Genetic factors have been increasingly considered to play an important role in the pathogenesis of PTDM. This meta-analysis showed that gene polymorphisms of *TCF7L2* rs7903146, *KCNQ1* rs2237892, and *KCNJ11* rs5219 contributed to PTDM occurrence and development. The genetic variations of *TCF7L2* rs12255372, *SLC30A8* rs13266634, *PPARγ* rs1801282, *CDKN2A/B* rs10811661, *HHEX* rs1111875, and *IGF2BP2* rs4402960 SNPs were not found to be associated with PTDM risk.

Previous studies indicated that these nine gene SNPs were associated with T2DM. Many genes associated with T2DM have also been associated with an increased risk of PTDM. T2DM and PTDM were thought to share certain common pathophysiological processes. Impaired insulin secretion and increased insulin resistance have been suggested as mechanisms underlying the development of PTDM. One of the most intensively studied genes was *TCF7L2*. TCF7L2, a key component of the Wnt signaling pathway, is involved in the regulation of pancreatic *β*-cell proliferation, differentiation, and insulin secretion [6, 10]. Two common SNPs, rs7903146 and rs12255372, were located in *TCF7L2* introns 3 and 4, respectively. *TCF7L2* rs7903146 C/T emerged as the most common susceptible gene for T2DM in genome-wide association studies (GWAS) [2, 51]. Its association with PTDM has been well demonstrated in Asian (Indian and Korean), White, and Caucasian populations [6–12]. The T allele mutation at *TCF7L2* rs7903146 loci has been linked with impaired insulin secretion and hepatic insulin resistance. The results of the association between *TCF7L2* rs12255372G/T and PTDM remained conflicting [6, 11, 12]. *TCF7L2* rs7903146 and rs12255372 haplotype analyses did not reveal any significant association with PTDM [11].

KCNQ1 encodes a subunit of the voltage-gated K + channel. It is expressed in the pancreas and may help regulate the membrane potential of insulin-secreting cells and is involved in triggering and maintaining glucose-stimulated insulin secretion [25, 43]. Although this meta-analysis suggested the susceptibility of the most common *KCNQ1* rs2237892 SNP to PTDM, opposite effects of *KCNQ1* rs2237892 polymorphism have been discussed. Hwang et al. showed that *KCNQ1* rs2237892C/T, located in intron 15, was significantly associated with decreased risk of PTDM in both allelic and genotype models, suggesting a protective effect on the development of PTDM [20]. Kang et al. reported that the T allele of *KCNQ1* rs2237892 was correlated with a high risk of PTDM in an allele-specific manner [8].

The pooled analysis of KCNJ11 genes suggested its role in the pathogenesis of PTDM. ATP-sensitive potassium channel KCNJ11 plays an important role in the regulation of insulin secretion by pancreatic *β* cells, as well as glucose metabolism. *KCNJ11* rs5219 glutamic acid to lysine amino acid substitution reduces potassium channels' sensitivity to ATP molecules, resulting in overactivity of the channel and subsequent inhibition of insulin secretion [12, 24, 25]. The meta-analysis of the Asian Indian population showed no significant association of *KCNJ11* rs5219 polymorphism with risk of T2DM [52]. However, other meta-analyses demonstrated a significant effect of *KCNJ11* rs5219 in susceptibility to T2DM in East Asians, Caucasians, and North Africans [53].

Controversial results have been reported for the association of SLC30A8, PPAR*γ*, CDKN2A/B, HHEX, and IGF2BP gene polymorphisms with PTDM. In this overall analysis, these extensively evaluated genes were not found to contribute to the development of PTDM. SLC30A8 belongs to the zinc transporter family, which plays a major role in transporting zinc from the cytoplasm to intracellular vesicles for insulin maturation, storage, and secretion from *β*-cells [7, 8, 10, 22, 38, 50]. The SLC30A8 rs13266634 arginine to tryptophan variant, associated with impaired *β*-cell function, has been proposed as important genetic markers of T2DM in Europeans and East Asians but not the African population [54, 55]. PPAR*γ* gene belongs to the nuclear hormone receptor subfamily that controls the expression of genes involved in glucose and lipid homeostasis. The SNP rs1801282 (C/G) is the most common variant located in exon-2 of *PPARγ*, and the substitution of proline to alanine of PPAR*γ* reduces its transcriptional activity and insulin sensitivity [7, 12, 21, 38, 41]. One meta-analysis suggested that *PPARγ* rs1801282 was significantly associated with T2DM under the heterozygote genetic model in Asian and Caucasian populations [56]. CDKN2A/B, which encodes two kinase inhibitors p16INK4a and p15INK4b, regulates pancreatic *β*-cell regeneration. The locus rs10811661 locates ∼100 kb upstream of CDKN2A/B gene-coding sequence, but the mechanism by which this SNP affects T2DM and PTDM susceptibility remains to be investigated [7, 8, 22, 38]. HHEX gene encodes a transcription factor involved in hepatic and pancreatic development via the Wnt signal pathway [7, 8, 22, 38]. The SNP rs1111875 at the 3′-flanking region of the HHEX gene, which may decrease pancreatic beta-cell function, is reported to be associated with T2DM risk as lead SNP in Chinese Han and European populations [57]. The meta-analysis of *IGF2BP2* rs4402960 suggested a significant association with T2DM in Asian populations [58]. The mRNA-binding protein IGF2BP2 is highly expressed in pancreatic islets and participates in a spectrum of the biological process including cellular metabolism. Rs4402960, located in the intron 2 region of *IGF2BP2*, has been found to attenuate glucose-stimulated insulin secretion [7, 8, 22, 38].

McCaughan et al. examined in GWAS the association between PTDM and 26 gene SNPs in the White population [59]. This association was validated for eight SNPs, and *KCNJ11* rs5219, *PPARγ* rs1801282, *SLC30A8* rs13266634, and *TCF7L2* rs7903146 polymorphisms were included, whereas the genetic variants of *TCF7L2* rs12255372, *KCNQ1* rs2237892, *CDKN2A/B* rs10811661, *HHEX* rs1111875, and *IGF2BP2* rs4402960 were not studied. These GWAS revealed the most significantly associated pathway of *β*-cell apoptosis and dysfunction in the pathogenesis of PTDM.

The previous meta-analysis by Benson et al. collected case-control kidney transplant studies that were carried out in Asian, Caucasian, and mixed ethnicity populations up to 2015 and investigated the association between 18 genetic variants across 12 genes and PTDM in the allele model [14]. They found *TCF7L2* rs7903146 and *KCNQ1* rs2237892 were correlated with higher PTDM risk, whereas the allelic distribution of *TCF7L2* rs12255372, *SLC30A8* rs13266634, *PPARγ* rs1801282, *CDKN2A/B* rs10811661, *HHEX* rs1111875, *IGF2BP2* rs4402960, and *KCNJ11* rs5219 was not linked with PTDM. Our meta-analysis included a number of updated publications till 2019, covering Asian, Caucasian, White, and African populations from both cohort and case-control studies. We comprehensively analyzed nine SNPs of eight genes in five allelic and genotype models, each model containing a minimum of three publications with complete data information, which would provide better power to identify alleles associated with PTDM susceptibility robustly. However, our study suggested *KCNQ1* rs2237892 was correlated with lower PTDM risk in the allele model. Furthermore, significant associations with PTDM were found for *TCF7L2* rs7903146 in the dominant, recessive, homozygote, and heterozygote genotype models; for *KCNQ1* rs2237892 in the dominant and heterozygote models; and for *KCNJ11* rs5219 in the recessive model. The meta-analysis by Quaglia et al. focused on *TCF7L2* rs7903146 studies published from 2009 to 2014 and showed that *TCF7L2* rs7903146 was strongly associated with PTDM in the dominant and recessive models, which was similar to our findings [13]. Moreover, both previous meta-analyses retrieved data from both candidate gene and GWAS on PTDM, whereas our study only incorporated studies based on the candidate gene method.

GWAS have identified more than 120 genetic loci associated with T2DM susceptibility [60]. In addition, many SNPs have been reported in candidate gene studies with T1DM and T2DM. The genetic variants predisposing to DM were commonly evaluated in PTDM development. Transcription factor encoding gene *HNF4A* [12], genes encoding renin-angiotensin system (RAS) including *ACE* and *AGT* [35, 44]; insulin-resistance genes of VDR (*Fox1*) [33], *adiponectin* [34, 40], and *PAI-1* [46]; insulin-sensitive gene *IRS* [12, 31]; glucose homeostasis genes *CAPN10* [47], *PPARα*, and *POR* [32, 36]; and inflammatory factor genes such as *CCL5* [34, 48], *IL-6* [37], *IL-1B*, *IL-2*, *IL-4*, *IL-17*, *IL-7R,* and *IL-17R* [18, 29, 39] have been shown to contribute to the pathogenesis of PTDM. Lower GPX1 enzyme activity, caused by *GPX1* 599C to T mutation, increases the exposure of pancreatic *β* cells to oxidative stress and development of PTDM [24, 49]. Additionally, *ATF6*, *GST* (*SOD* and *CAT*), *INFγ* and (*TGFβ1*, *TNFα*, and *STAT4*) polymorphisms, which play important roles in endoplasmic reticulum stress, oxidative stress, and inflammation respectively, were not found to be associated with PTDM [16, 28, 41, 42, 45, 49]. In recent studies, new evidence have suggested that genetic variants of TAC metabolizing enzymes including *CYP3A4* and *CYP24A1* were associated with increased risk of PTDM [21, 23]. *GCK*, *LEP*, *LEPR*, and *PCK2* SNPs may contribute to PTDM by influencing glucose and lipid homeostasis [19, 22, 23, 30]. Another ATP-sensitive potassium channel gene *ABCC8* encoding SUR1 was implicated to be associated with a high prevalence of PTDM. Moreover, other inflammation genes including *TLR4*, *TLR6* [27], *MBL2* [18], transcription factor *HNF1β* [17], and matrix metalloproteinase gene *MMP-2* SNPs may also predispose transplant recipients to the development of PTDM. The effect size of several genetic variants, such as *GPX1* 599TT, *CYP24A1* rs2296241 AA, *IL-17F* rs763780TC, *LEP* rs2167270 AA, *PCK2* rs4982856TT, *TLR6* rs1039559 CC, and *MMP-2* rs1132896 CC are relatively large (ORs between 3.5 and 10) [21, 22, 26, 27, 29, 30, 49]. Furthermore, *IL-1B* rs3136558, *IL-2* rs2069762, *IL-7R* rs1494558, *IL-7R* rs2172749, *IL-17R* rs2229151, *IL-17R* rs4819554 [39], *MMP-2* rs243849 [26], *IL-6* 174 [37], *TLR4* rs1927914 [27], *PAI-1* −675 5G5G [46], and *CAPN10* SNP-63 rs5030952 [47] were reported to confer protective effects for the development of PTDM. However, the number of studies for these reported gene polymorphisms was limited. There were only one or two relevant articles available, which could not provide enough statistical power to detect differences in the incidence of PTDM between different genotype groups. The association between these gene SNPs and PTDM susceptibility was still inconclusive and further exploration was needed.

This study had several limitations. First, the etiopathogenesis of PTDM was multifactorial. Immunosuppressive regimen, ethnicity, older age, sex, BMI, and other related clinical characteristics contributed significantly to the risk of PTDM. However, crude estimates of effect were often used to evaluate the association between genes polymorphisms and PTDM without adjustments for other confounding variables. Second, PTDM in kidney recipients occurred mainly during the first months. Additionally, there could be a reversible phenotype change from PTDM to non-PTDM. In this study, there was high heterogeneity regarding the observational follow-up time after renal transplantation, which varied from 3 to 12 months among the studies. Third, treatment modality varied greatly for different studies, which may substantially influence the overall incidence of PTDM. Fourth, certain minor allele frequencies (MAF) differed greatly in different races. The sample size in some studies might be too small to detect minor effects, and some study populations presented with various genetic backgrounds. Furthermore, for most studies, it is unclear whether there was preexisting impaired glucose tolerance, which may affect the estimated incidence of PTDM.

Our meta-analysis revealed a significant association between PTDM and gene polymorphisms of *TCF7L2* rs7903146, *KCNQ1* rs2237892, and *KCNJ11* rs5219. Furthermore, we reviewed the literature on available gene SNPs that were susceptible to PTDM. The regulatory mechanism of relevant genes SNPs in the occurrence and development of PTDM was worthy of further exploration. SNPs showing association may serve as genetic markers for the prediction of the development of PTDM, combined with other risk factors of PTDM. Alternate medication of diabetogenic drugs may be considered for early prevention of PTDM based on risk assessment. Further large sample studies with diverse race populations are necessary to confirm our findings.

## Figures and Tables

**Table 1:** Characteristics of the included studies.

Study ID	CountryEthnicity	Design	Genotyping methods	Immunosuppressive treatment	Diagnostic criteria of cases	Time of PTDM diagnosis after transplantation (months)	Sample size	Age at transplantation (mean ± SD), *y*	Gender female (%)
							PTDM/non-PTDM	PTDM/non-PTDM	PTDM/non-PTDM
Van der Burgh [17]	Netherlands	Prospective cohort	PCR	TAC	ADA criteria	12	29/138	60 ± 7/51 ± 15	34.5/41.3
Guad [18]	Malaysia/Malay, Chinese, Indian	Cohort	PCR	CSA/TAC/both	ADA criteria	12	29/139	39.3 ± 13.4/33.9 ± 11.8	44.8/40
Mota-Zamorano [19]	Spain/Caucasian	Cohort	RT-PCR	CSA/TAC	ADA criteria	12	57/258	—	—
Hwang [20]	Korean/	Prospective, multicenter, nationwide cohort study	PCR	TAC/steroid	ADA criteria	12	254/848	52.2 ± 10.4/45.1 ± 12.0	40.2/47.5
Zhang [21]	China/Chinese, Han	Cohort	PCR-RFLP	Triple-therapy/TAC, MMF, steroid	ADA criteria	6	17/112	49.35 ± 9.06/46.56 ± 9.91	29.4/23.2
Yokoyama [22]	Japan/Japanese	Cohort	PCR	CSA/TAC		12	11/27	37.3 ± 9.0/44.6 ± 15.0	27.2/44.4
Shi [23]	China/Chinese, Han	Case-control	PCR	TAC	ADA criteria	3	57/112	43.1 ± 9.0/38.6 ± 11.8	—
Yalin [24]	Turkey	Monocenter case-control	PCR-RFLP	CSA + AZA + PRED/CSA + MMF + PRED/TAC + MMF + PRED	ADA criteria	—	58/60	47.2 ± 11.0/38.5 ± 10.1	31/36.7
Dabrowska-Zamojcin [25]	Poland	Cohort	RT-PCR	Standard triple-therapy TAC, MMF, and steroids	ADA criteria	8.6	35/166	—	—
Alagbe [6]	South Africa	Cohort	PCR	CSA/TAC	ADA criteria	12 (TAC)/36 (CSA)	20/91	44/37	37.4/50
Ong [26]	Korea	Cohort	PCR	TAC/others	ADA criteria		52/257	45.11 ± 9.90/38.26 ± 11.17	46.4/39.2
Kim [27]	Korea	Cohort	PCR	CSA/TAC/others	ADA criteria	3	51/254	45.56 ± 1.28/38.28 ± 0.71	47.1/39.4
Dabrowska-Zamojcin [28]	Poland	Cohort	RT-PCR	Triple-drug therapy, CSA/TAC, AZA or MMF, and steroids	ADA criteria	3	23/146	—	—
Romanowski [29]	Poland/Caucasian	Cohort	RT-PCR	TAC/CSA	ADA criteria	3	43/272	—	—
Romanowski [30]	Poland/Caucasian	Cohort	RT-PCR	Triple-therapy TAC, MMF, and steroids	ADA criteria	3	23/146	—	—
Khan [10]	India	Cohort	PCR-RFLP	CSA/TAC	ADA criteria	3	42/98	39.57 ± 11.8/39.48 ± 10.59	28.6/23.5
Chen [31]	China/Chinese	Cohort	PCR	TAC	WHO guidelines	1	78/80	40.4 ± 9.4/38.7 ± 8.2	25.6/26.3
Kurzawski [32]	Poland/White	Cohort	RT-PCR	TAC	ADA criteria	12	48/176	—	—
Yao [33]	China/Chinese	Cohort	PCR-RFLP	MMF and corticosteroids	ADA criteria	6	16/89	47.81 ± 15.54/36.62 ± 11.43	37.5/34.8
Nicoletto [34]	Brazil/Caucasian	Cohort	RT-PCR	CSA/TAC	ADA criteria	12	83/187	48.1 ± 11.0/39.8 ± 11.9	39.6/39.8
Lee [35]	Korea	Cohort	PCR	TAC/others	ADA criteria	3	49/253	45.18 ± 9.39/38.1 ± 11.21	46.9/38.7
Elens [36]	Belgium	Cohort	RT-PCR	TAC	—	—	9/76	—	—
Weng [37]	China/Taiwan	Cohort	PCR-RFLP	CSA/TAC	International consensus guidelines	—	27/251	47.6 ± 9.8/41.7 ± 11.5	44.6/22.2
Kurzawski [38]	Poland/Caucasian	Cohort	RT-PCR	TAC	ADA criteria	12	67/168	47.7 ± 10.6/43.2 ± 13.0	45.5/46.4
Kim [39]	Korea	Cohort	PCR	TAC/others	ADA criteria	3	53/253	44.91 ± 1.33/38.34 ± 0.71	47.2/39.5
Kang [39]	Korea	Cohort	PCR	CSA/TAC	The International Consensus Guidelines	12	154/421	42.3 ± 9.2/37.3 ± 9.4	37.7/35.6
Yu [40]	China/Chinese	Cohort	PCR	CSA or TAC, mycophenolate or AZA, and steroid.	ADA criteria	24	97/301	45.55 ± 10.78/40.26 ± 11.47	19.6/33.9
Yang [12]	USA	Cohort	RT-PCR	CSA or TAC, mycophenolic acid derivatives, sirolimus, and PED	ADA criteria		133/170	44.30 ± 13.79/41.01 ± 13.11	43.6/43.5
Wang [41]	UAS/White, African American, Hispanic, Asian	Case-control	PCR	TAC and MMF	ADA criteria	3	51/72	49.02 ± 13.04/47.22 ± 12.83	45.1/37.5
Tsai [42]	China/Taiwan	Cohort	PCR-RFLP	TAC	ADA criteria	19.27 ± 26.3	85/198	54.9 ± 9.36/50.6 ± 11	45.9/50
Tavira [43]	Spain/Caucasian	Cohort	PCR-RFLP	Standard triple TAC, MMF, and PED	ADA criteria	12	145/260	49 ± 11/44 ± 13	40/38
Özdemir [44]	Turkey	Cohort	PCR	Standard triple therapy with TAC, MMF, and PED	ADA criteria/WHO guidelines	12	23/27	37.9 ± 10.5/38.3 ± 10.9	33.3/35
Kurzawski [11]	Poland	Cohort	RT-PCR	TAC, MMF, and steroids	ADA criteria	12	66/168	47.7 ± 10.6/43.2 ± 13.0	45.5/46.4
Fougeray [45]	France/Caucasians, Black, Asiatics, Other/unknown	Cohort	PCR	TAC and MMF	ADA criteria	3	21/248	—	—
Chang [46]	China/Taiwan	Cohort	PCR-RFLP	CSA or TAC, MMF, or mycophenolic acid with or without PED	ADA criteria	Any time in follow-up	81/259	55.3 ± 10.0/52.6 ± 11.3	43.2/48.4
Kurzawski [47]	Poland	Cohort	PCR	TAC, MMF, and steroids	ADA criteria	12	56/158	47.3 ± 9.9/43.0 ± 13.2	51.8/52.5
Kao [16]	China/Taiwan	Cohort	PCR-RFLP	CsA/FK506	ADA criteria	Any time in follow-up	73/241	49.4 ± 9.37/47 ± 10.85	42.5/47.3
Jeong [48]	Korea	Cohort	PCR	TAC/others	ADA criteria	3	56/255	45.11 ± 9.90/38.26 ± 11.17	46.4/39.2
Dutkiewicz [49]	Poland/Caucasian	Cohort	PCR-RFLP	TAC, MMF, and steroids	ADA criteria	3	21/138	46.8 ± 8.8/42.0 ± 13.6	33.3/43.5
Kang [8]	Korea	Cohort	PCR	Calcineurin inhibitors and GC	International consensus guidelines	12	145/444	42.6 ± 9.1/37.4 ± 9.3	35.2/34.7
Ghisdal [7]	France	Cohort	RT-PCR	CSA/TAC/mTOR inhibitor	ADA criteria	6	118/958	52.8/46.7	42.4/37.1
Kang [9]	Korea	Cohort	PCR	CSA/TAC	ADA criteria	3	174/450	42.1 ± 8.99/35.42 ± 9.43	35.1/35.6
Kang [50]	Korea	Cohort	RT-PCR	CSA/TAC and GC	ADA criteria	3	119/391	41.10 ± 9.33/35.64 ± 10.8	34.5/36.5

PCR: polymerase chain reaction; RT-PCR: real-time polymerase chain reaction; PCR-RFLP: polymerase chain reaction-restriction fragment length polymorphism; ADA: American Diabetes Association; WHO: World Health Organization; CSA: cyclosporine A; AZA: azathioprine; PRED: prednol; MMF: mycofenolat mophetil; TAC: tacrolimus; PED: prednisone; PTDM: posttransplant diabetes mellitus; and GC: glucocorticoids.

**Table 2:** Quality assessment.

Study	Representativeness of the exposed cohort	Selection of the non-exposed cohort	Ascertainment of exposure	Demonstration that outcome of interest was not present at the start of the study	Comparability of cohorts on the basis of the design or analysis	Assessment of outcome	Was follow-up long enough for outcomes to occur	Adequacy of follow-up of cohorts	Total quality scores
**Cohort**									
Van der Burgh [17]	*∗*	*∗*	*∗*	*∗*	*∗∗*	*∗*	*∗*	*∗*	9
Guad [18]	*∗*	*∗*	*∗*	*∗*	*∗∗*	*∗*	*∗*	*∗*	9
Mota-Zamorano [19]	*∗*	*∗*	*∗*	*∗*	*∗*	*∗*	*∗*	*∗*	8
Hwang [20]	*∗*	*∗*	*∗*	*∗*	*∗∗*	*∗*	*∗*	*∗*	9
Zhang [21]	*∗*	*∗*	*∗*	*∗*	*∗∗*	*∗*	*∗*	*∗*	9
Yokoyama [22]	*∗*	*∗*	*∗*	*∗*	*∗∗*	*∗*	*∗*	*∗*	9
Shi [23]	*∗*	*∗*	*∗*	*∗*	*∗*	*∗*	*∗*	*∗*	8
Dabrowska-Zamojcin [25]	*∗*	*∗*	*∗*	*∗*	*∗*	*∗*	*∗*	*∗*	8
Alagbe [6]	*∗*	*∗*	*∗*	—	*∗∗*	*∗*	*∗*	*∗*	8
Ong [26]	*∗*	*∗*	*∗*	*∗*	*∗*	*∗*	—	—	6
Kim [27]	*∗*	*∗*	*∗*	*∗*	*∗*	*∗*	*∗*	*∗*	8
Dabrowska-Zamojcin [28]	*∗*	*∗*	*∗*	*∗*	—	*∗*	*∗*	*∗*	7
Romanowski [29]	*∗*	*∗*	*∗*	*∗*	—	*∗*	*∗*	*∗*	7
Romanowski [30]	*∗*	*∗*	*∗*	*∗*	*∗*	*∗*	*∗*	*∗*	8
Khan [10]	*∗*	*∗*	*∗*	*∗*	*∗∗*	*∗*	—	—	7
Chen [31]	*∗*	*∗*	*∗*	*∗*	*∗∗*	*∗*	*∗*	*∗*	9
Kurzawski [32]	*∗*	*∗*	*∗*	*∗*	*∗∗*	*∗*	*∗*	*∗*	9
Yao [33]	*∗*	*∗*	*∗*	*∗*	*∗*	*∗*	*∗*	*∗*	8
Nicoletto [34]	*∗*	*∗*	*∗*	*∗*	*∗*	*∗*	*∗*	*∗*	8
Lee [35]	*∗*	*∗*	*∗*	*∗*	*∗∗*	*∗*	*∗*	*∗*	9
Elens [36]	*∗*	*∗*	*∗*	*∗*	—	*∗*	*∗*	—	6
Weng [37]	*∗*	*∗*	*∗*	*∗*	*∗*	*∗*	*∗*	*∗*	8
Kurzawski [38]	*∗*	*∗*	*∗*	*∗*	*∗∗*	*∗*	*∗*	*∗*	9
Kim [39]	*∗*	*∗*	*∗*	*∗*	*∗∗*	*∗*	*∗*	*∗*	9
Kang [8]	*∗*	*∗*	*∗*	*∗*	*∗∗*	*∗*	*∗*	*∗*	9
Yu [40]	*∗*	*∗*	*∗*	*∗*	*∗∗*	*∗*	*∗*	*∗*	9
Yang [12]	*∗*	*∗*	*∗*	*∗*	*∗∗*	*∗*	*∗*	*∗*	9
Tsai [42]	*∗*	*∗*	*∗*	*∗*	*∗*	*∗*	*∗*	*∗*	8
Tavira [43]	*∗*	*∗*	*∗*	*∗*	*∗∗*	*∗*	*∗*	*∗*	9
Özdemir [44]	*∗*	*∗*	*∗*	*∗*	*∗∗*	*∗*	*∗*	*∗*	9
Kurzawski [11]	*∗*	*∗*	*∗*	*∗*	*∗*	*∗*	*∗*	*∗*	8
Fougeray [45]	*∗*	*∗*	*∗*	*∗*	—	*∗*	*∗*	*∗*	7
Chang [46]	*∗*	*∗*	*∗*	*∗*	*∗*	*∗*	*∗*	*∗*	8
Kurzawski [47]	v	*∗*	*∗*	*∗*	*∗∗*	*∗*	*∗*	*∗*	9
Kao [16]	*∗*	*∗*	*∗*	*∗*	*∗∗*	*∗*	*∗*	*∗*	9
Jeong [48]	*∗*	*∗*	*∗*	*∗*	*∗*	*∗*	*∗*	*∗*	8
Dutkiewicz [49]	*∗*	*∗*	*∗*	*∗*	*∗∗*	*∗*	*∗*	*∗*	9
Kang [8]	*∗*	*∗*	*∗*	*∗*	*∗∗*	*∗*	*∗*	*∗*	9
Ghisdal [7]	*∗*	*∗*	*∗*	*∗*	*∗∗*	*∗*	*∗*	*∗*	9
Kang [9]	*∗*	*∗*	*∗*	*∗*	*∗∗*	*∗*	*∗*	*∗*	9
Kang [50]	*∗*	*∗*	*∗*	*∗*	*∗∗*	*∗*	*∗*	*∗*	9
**Case-control**									

Study	Is the case definition adequate?	Representativeness of the cases	Selection of controls	Definition of controls	Comparability of cases and controls on the basis of the design or analysis	Ascertainment of intervention	Same method of ascertainment for cases and controls	Non-response rate	Total quality scores
Yalin [24]	*∗*	*∗*	*∗*	*∗*	*∗∗*	*∗*	*∗*	*∗*	9
Wang [41]	*∗*	*∗*	*∗*	*∗*	*∗∗*	*∗*	*∗*	*∗*	9

^∗^One point; ^∗∗^two points.

**Table 3:** Genetic polymorphisms and risk of PTDM after renal transplantation.

	Model	No. of paper	OR	95% CI	*P* value	*I* ^2^%	*P* value
							(Heterogeneity)
TCF7L2 (rs7903146)	Allele model	7	1.59	1.17–2.16	0.003	60.8	0.018
	Dominant model	7	1.62	1.14–2.31	0.007	54.6	0.040
	Heterozygote model	7	1.50	1.08–2.10	0.017	45.9	0.085
	Homozygote model	5	2.21	1.23–3.94	0.008	35.3	0.186
	Recessive model	5	1.87	1.18–2.94	0.007	12.8	0.332

TCF7L2 (rs12255372)	Allele model	3	0.16	0.87–1.54	0.314	0	0.407
	Dominant model	3	1.18	0.78–1.79	0.424	20.7	0.283
	Heterozygote model	3	1.15	0.74–1.81	0.529	24.8	0.991
	Homozygote model	3	1.45	0.70–3.00	0.317	0	0.925
	Recessive model	3	1.36	0.67–2.76	0.401	0	0.264

SLC30A8 (rs13266634)	Allele model	6	1.28	0.70–2.32	0.421	93.4	<0.001
	Dominant model	6	1.29	0.68–2.44	0.442	87.4	<0.001
	Heterozygote model	6	1.16	0.68–1.97	0.593	79.0	<0.001
	Homozygote model	6	1.66	0.52–5.30	0.396	90.9	<0.001
	Recessive model	6	1.43	0.55–3.72	0.467	89.6	<0.001

KCNQ1 (rs2237892)	Allele model	4	0.68	0.58–0.81	<0.001	0	0.473
	Dominant model	4	0.6	0.49–0.74	<0.001	0	0.717
	Heterozygote model	4	0.61	0.48–0.76	<0.001	0	0.890
	Homozygote model	4	0.75	0.35–1.58	0.444	59.6	0.059
	Recessive model	4	0.87	0.44–1.69	0.672	53.4	0.092

PPAR*γ* (rs1801282)	Allele model	5	0.98	0.75–1.28	0.885	0	0.642
	Dominant model	5	1.04	0.78–1.40	0.772	0	0.665
	Heterozygote model	5	1.11	0.82–1.48	0.505	0	0.713
	Homozygote model	3	0.44	0.12–1.61	0.217	0	0.93
	Recessive model	3	0.44	0.12–1.60	0.213	0	0.936

CDKN2A/B (rs10811661)	Allele model	4	1.10	0.79–1.52	0.588	52.8	0.095
	Dominant model	4	1.51	0.95–2.38	0.079	0	0.641
	Heterozygote model	4	1.54	0.96–2.48	0.075	0	0.877
	Homozygote model	4	1.52	0.93–2.49	0.092	0	0.462
	Recessive model	4	1.06	0.71–1.57	0.778	46.6	0.132

HHEX (rs1111875)	Allele model	4	1.15	0.89–1.50	0.283	45.3	0.139
	Dominant model	4	1.35	0.98–1.86	0.067	19.2	0.294
	Heterozygote model	4	1.35	1.00–1.84	0.051	7.2	0.357
	Homozygote model	4	1.30	0.74–2.30	0.357	46.8	0.130
	Recessive model	4	1.09	0.65–1.83	0.735	53.5	0.092

IGF2BP2 (rs4402960)	Allele model	4	0.97	0.78–1.21	0.801	20.0	0.290
	Dominant model	4	0.92	0.63–1.34	0.670	49.3	0.116
	Heterozygote model	4	0.88	0.57–1.36	0.559	55.5	0.081
	Homozygote model	4	1.14	0.76–1.71	0.532	0	0.663
	Recessive model	4	0.23	0.83–1.82	0.292	0	0.692

KCNJ11 (rs5219)	Allele model	3	1.10	0.74–1.63	0.651	56.3	0.102
	Dominant model	3	0.98	0.57–1.66	0.929	50.1	0.135
	Heterozygote model	3	0.90	0.58–1.40	0.641	20.1	0.286
	Homozygote model	3	1.45	0.79–2.66	0.228	21.5	0.280
	Recessive model	3	1.59	1.01–2.50	0.047	0	0.575

## Data Availability

Since it is a meta-analysis, all data were extracted from public databases, and all data were available in Supplementary Materials.

## Supplementary Materials

Supplementary Materials The supplementary materials contain supplementary figures and tables and PRISMA checklist.
