# Supplementary material for: Association between Genetic Polymorphisms and Risk of Kidney Posttransplant Diabetes Mellitus: A Systematic Review and Meta-Analysis
Source: Int J Clin Pract. 2022 Mar 8;2022:7140024. doi: 10.1155/2022/7140024 (PMC9159121; doi:10.1155/2022/7140024)
Supplement: Supplementary Materials — The supplementary materials contain supplementary figures and tables and PRISMA checklist. [file 7140024.f1.zip › 7140024.f1/Supplementary Figures.docx]

**Supplementary Figure 1-3 Sensitivity analysis**

**sFigure1**

1. **TCF7L2(rs7903146)-Allele model**

**
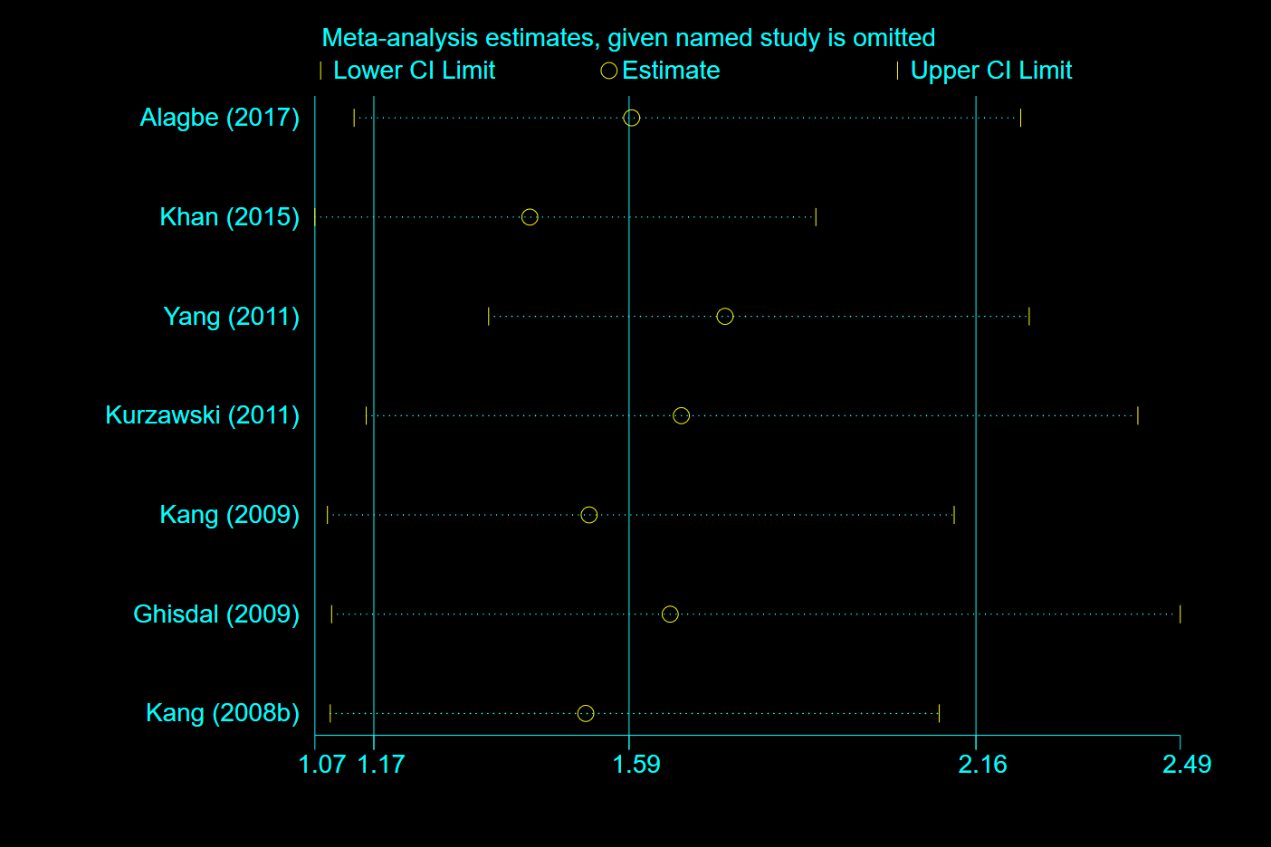
**

1. **TCF7L2(rs7903146)-Dominant model**

**
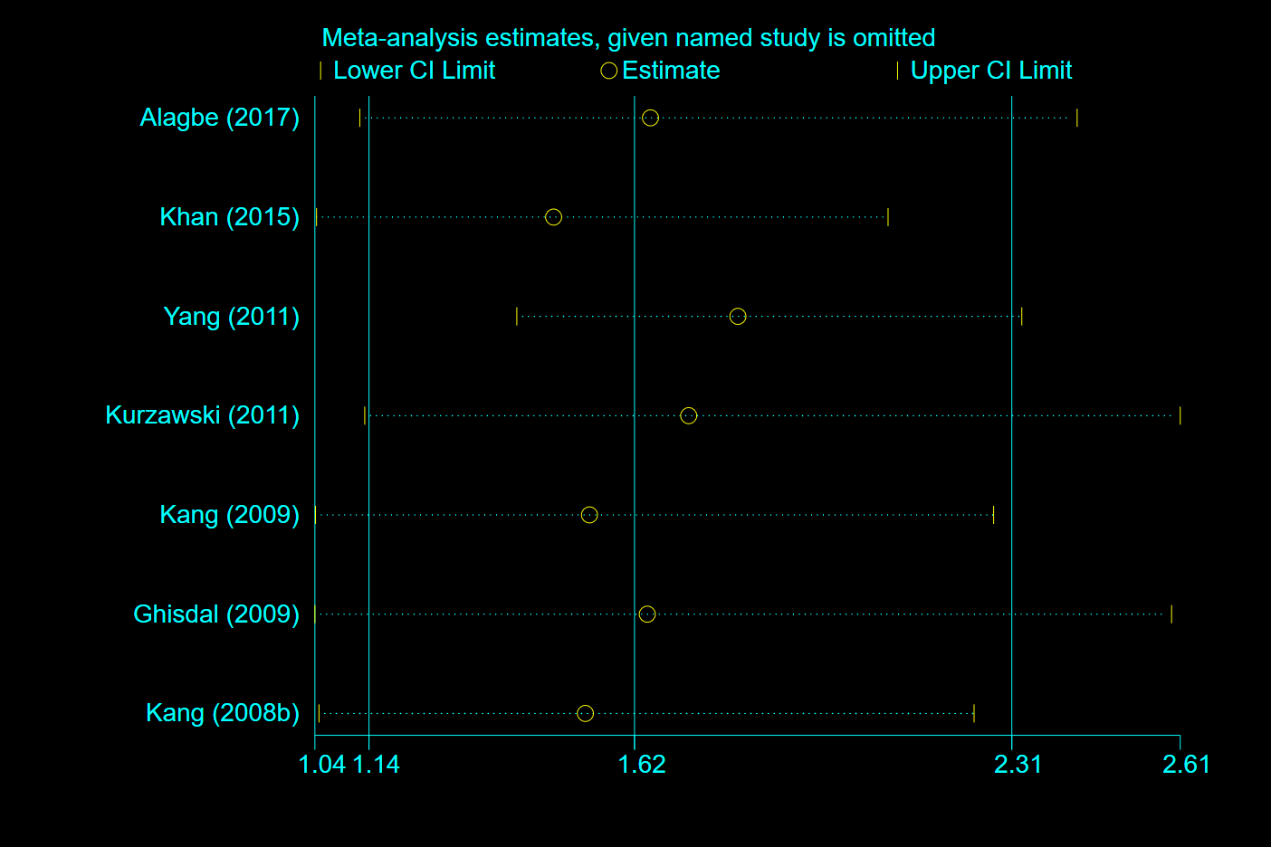
**

1. **TCF7L2(rs7903146)-Recessive model**

**
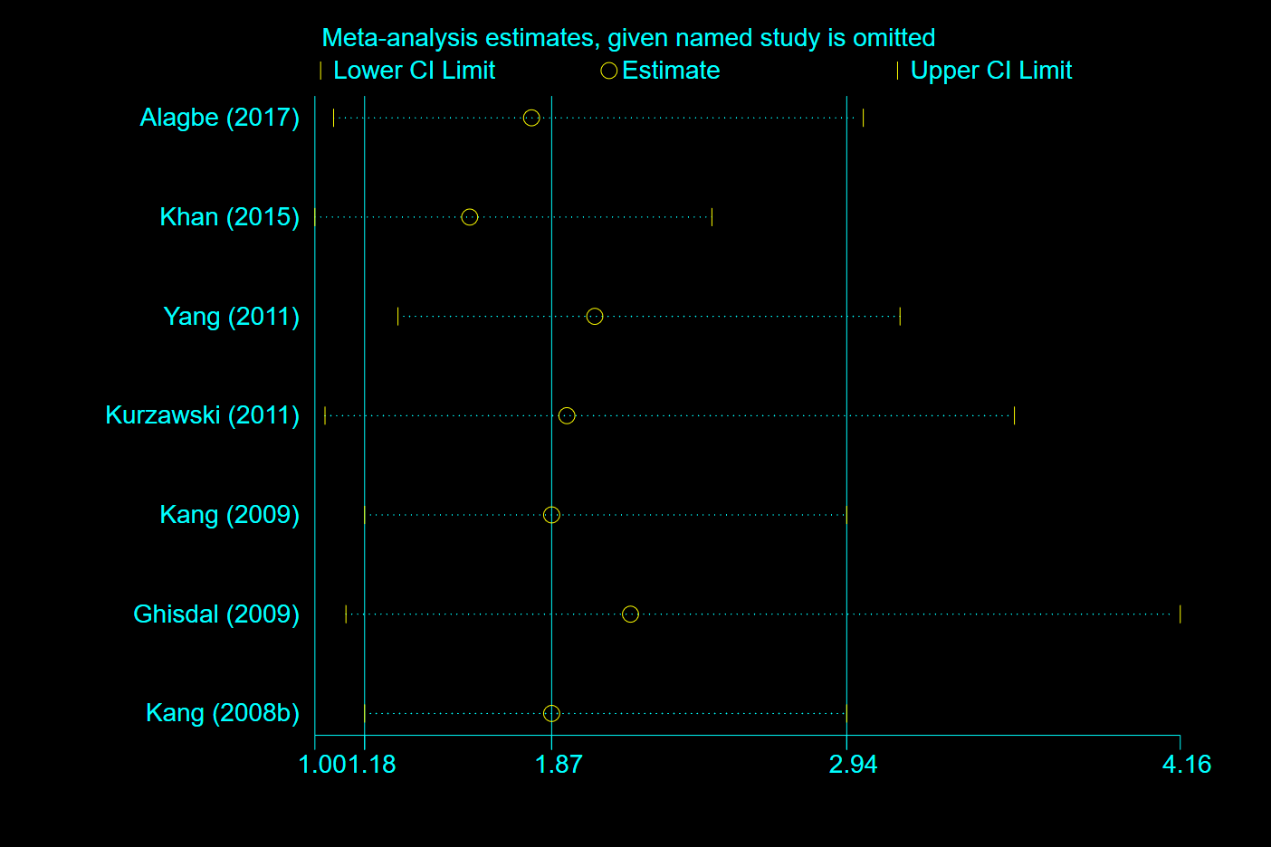
**

1. **TCF7L2(rs7903146)-Homozygote model**

**
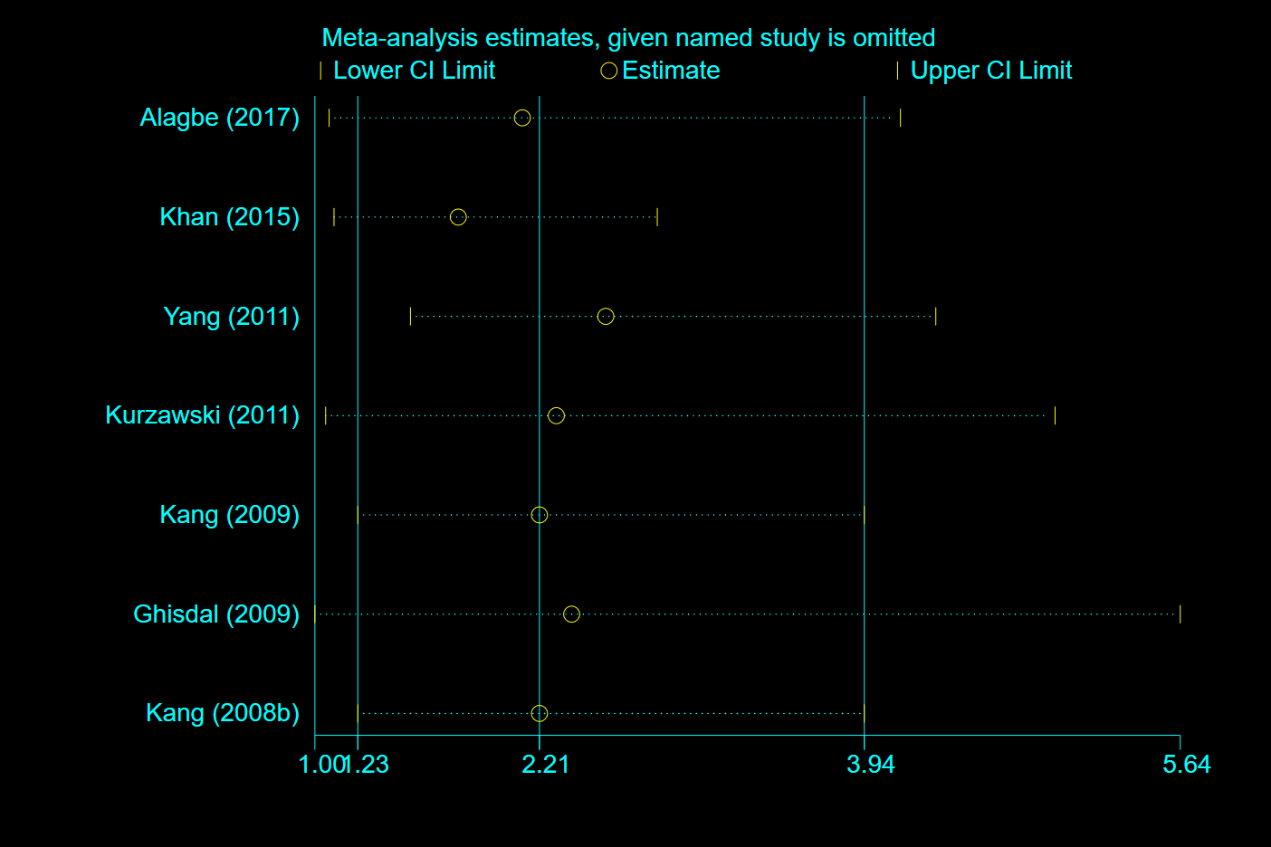
**

1. **TCF7L2(rs7903146)-Heterozygote model**

**
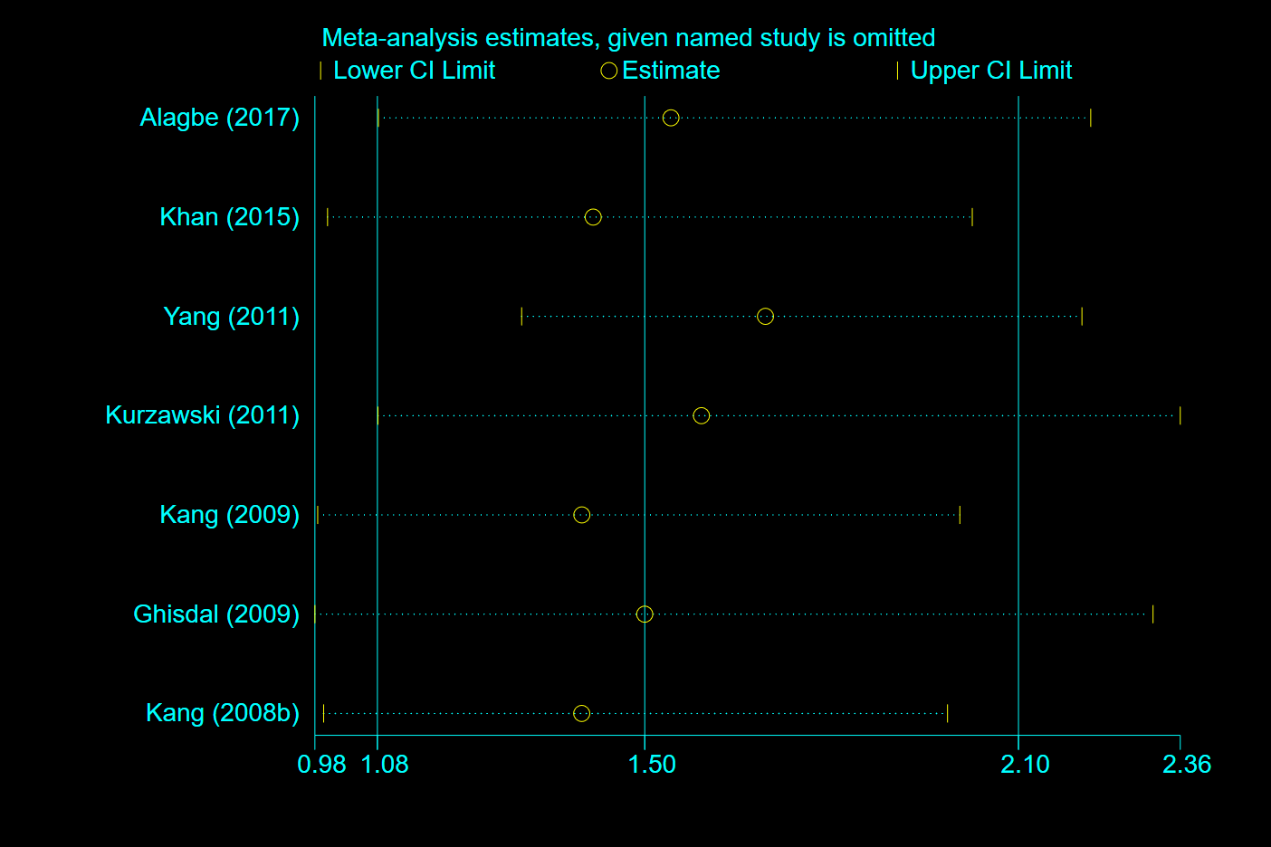
**

**sFigure2**

1. **SLC30A8(rs13266634)-Allele model**

**
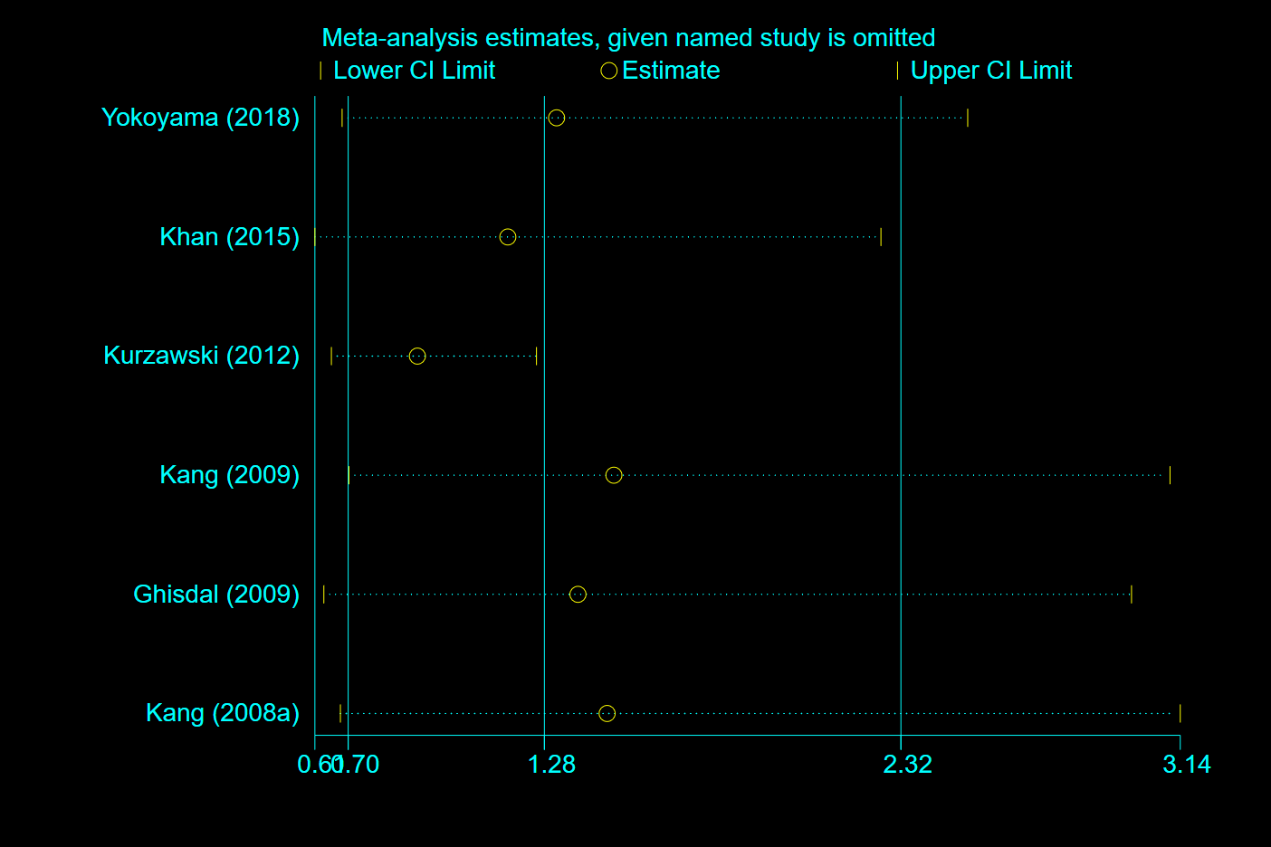
**

1. **SLC30A8(rs13266634)-Dominant model**

**
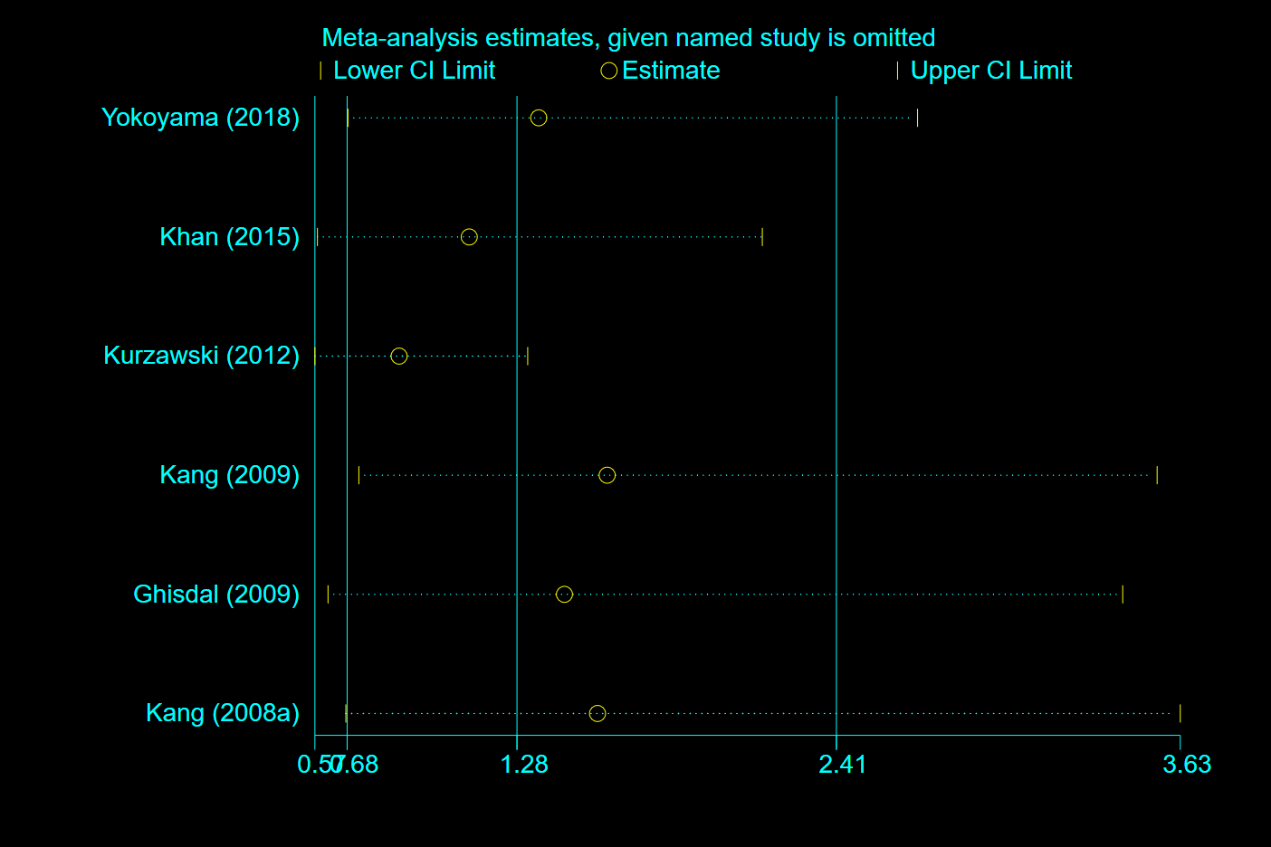
**

1. **SLC30A8(rs13266634)-Recessive model**

**
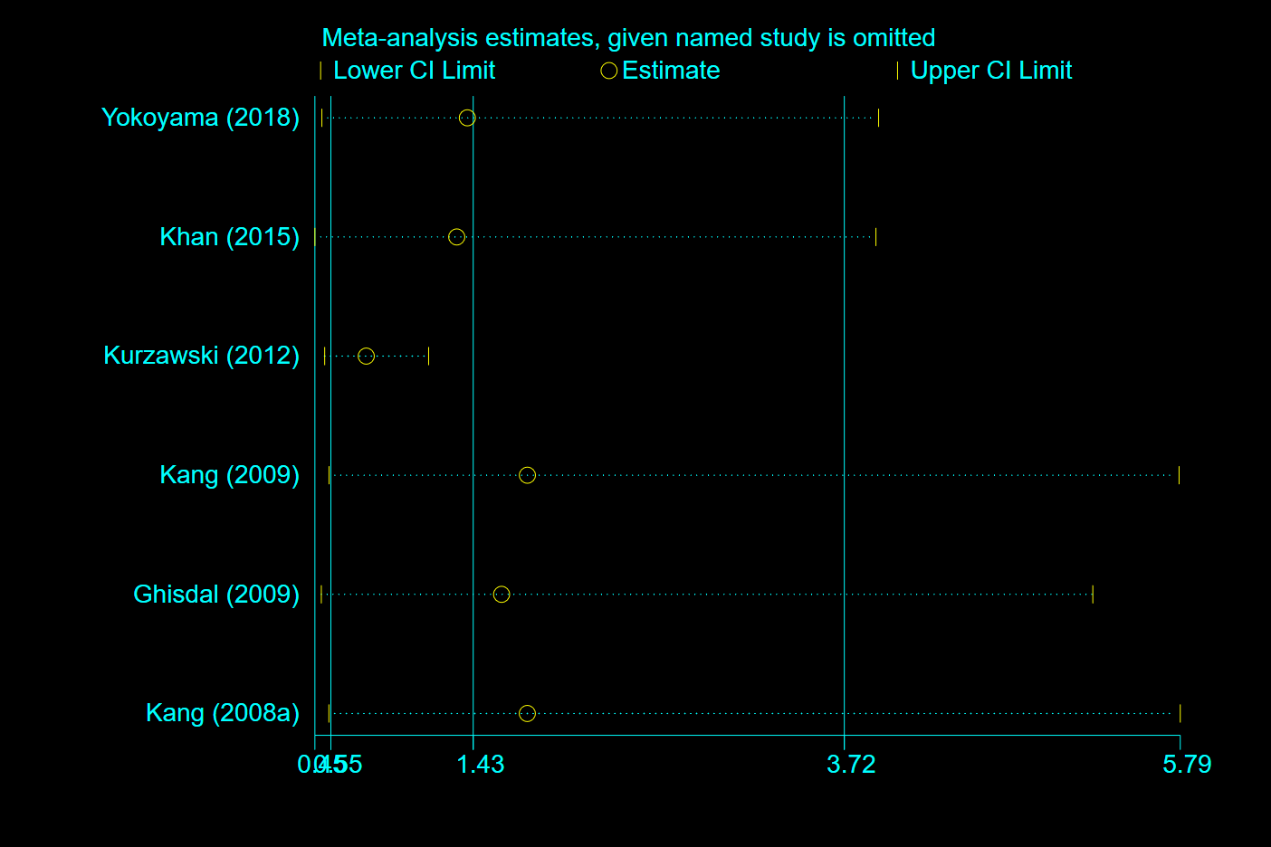
**

1. **SLC30A8(rs13266634)-Homozygote model**

**
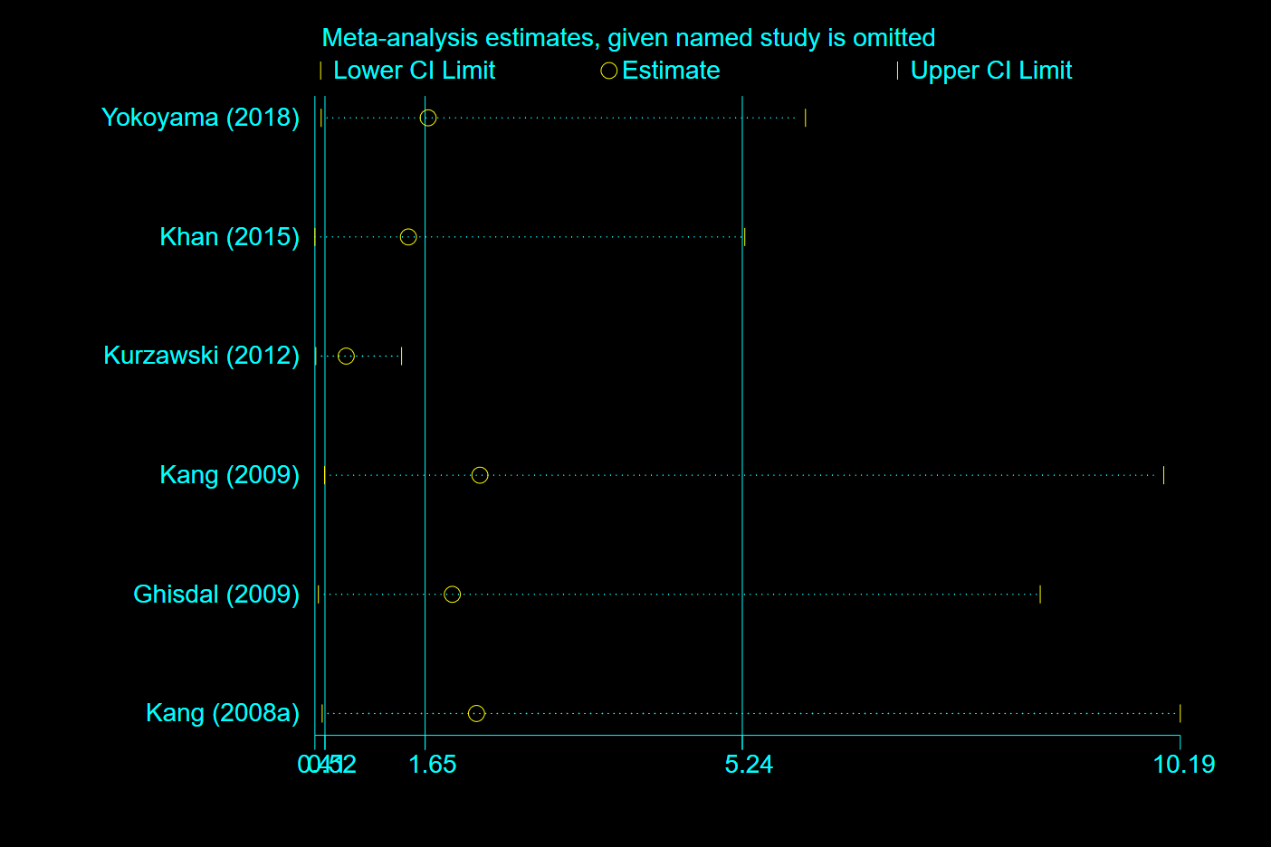
**

1. **SLC30A8(rs13266634)-Heterozygote model**

**
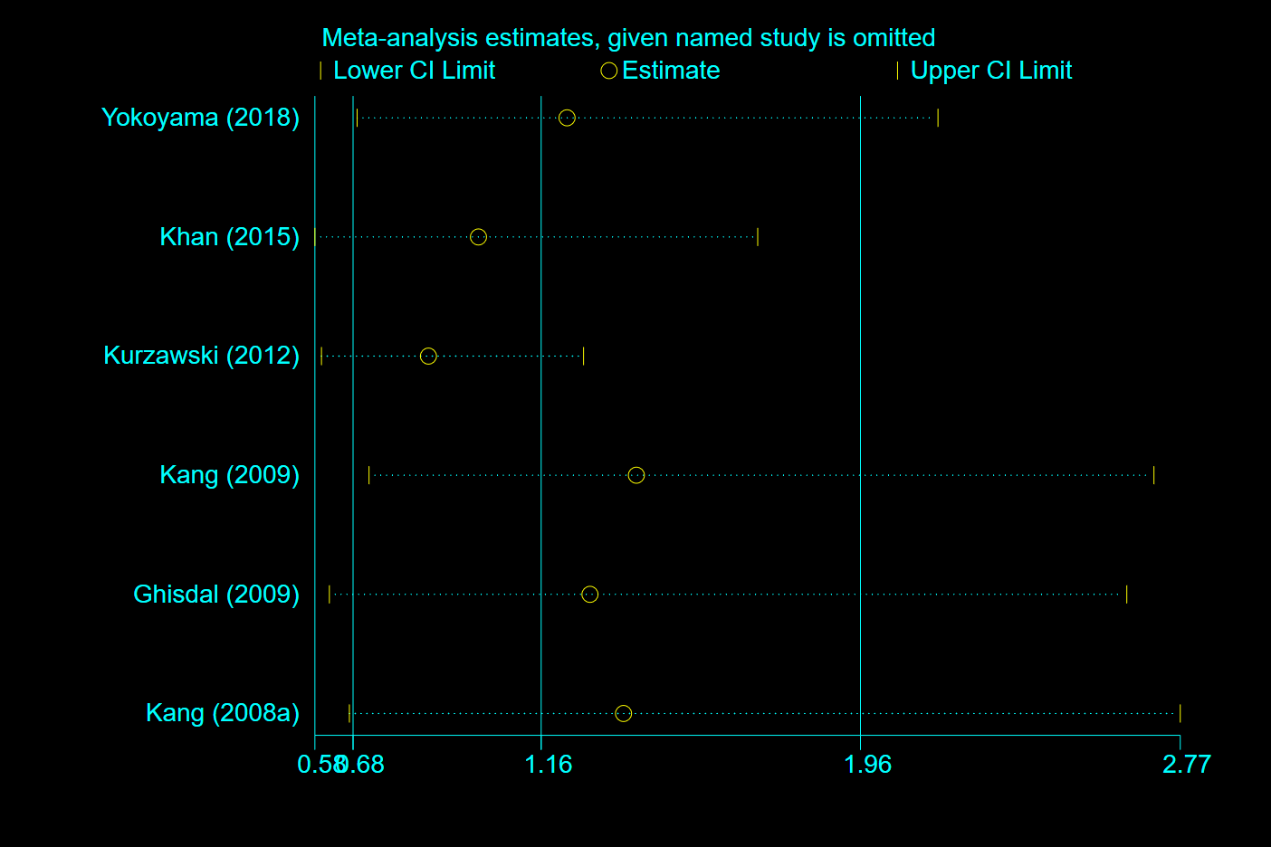
**

**sFigure3**

**PPARγ(rs1801282)-Allele model**

**
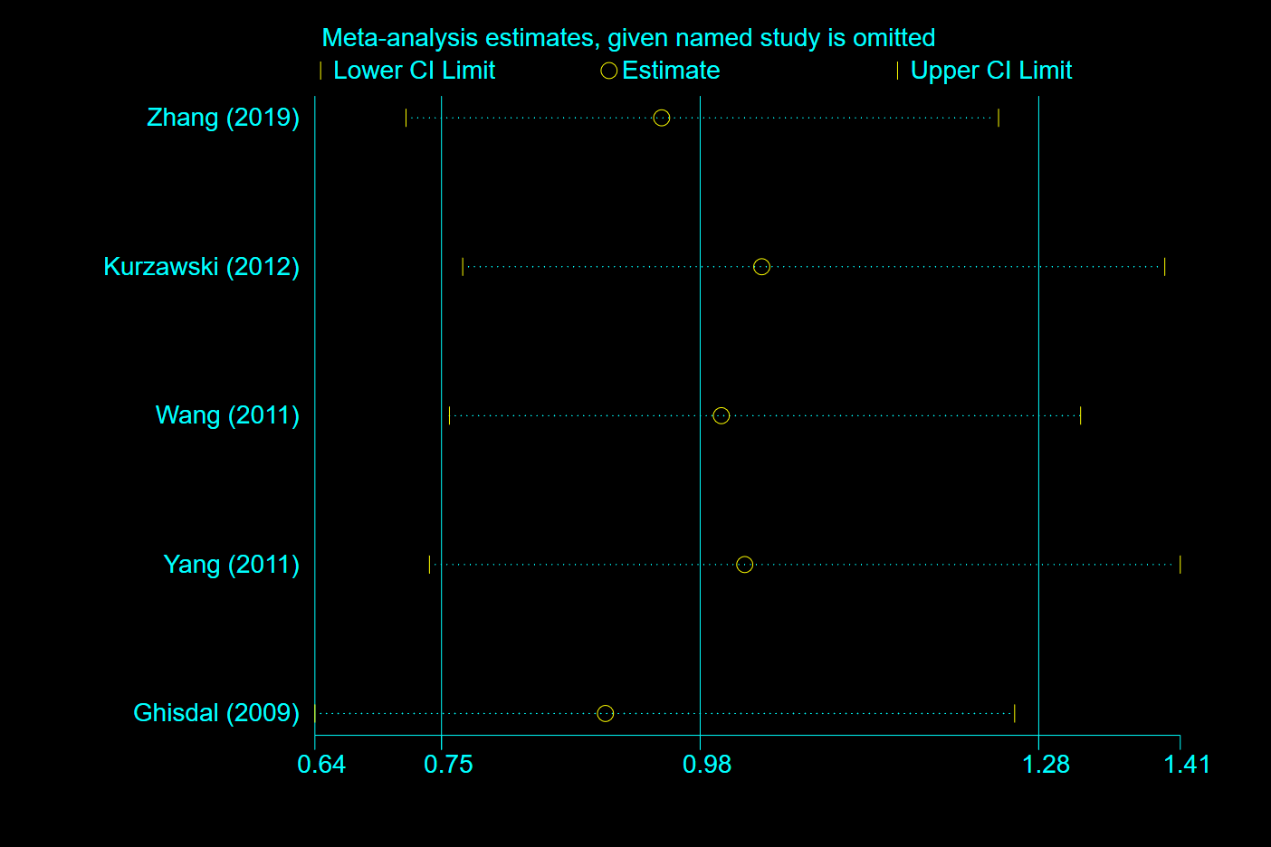
**

**PPARγ(rs1801282)-Dominant model**

**
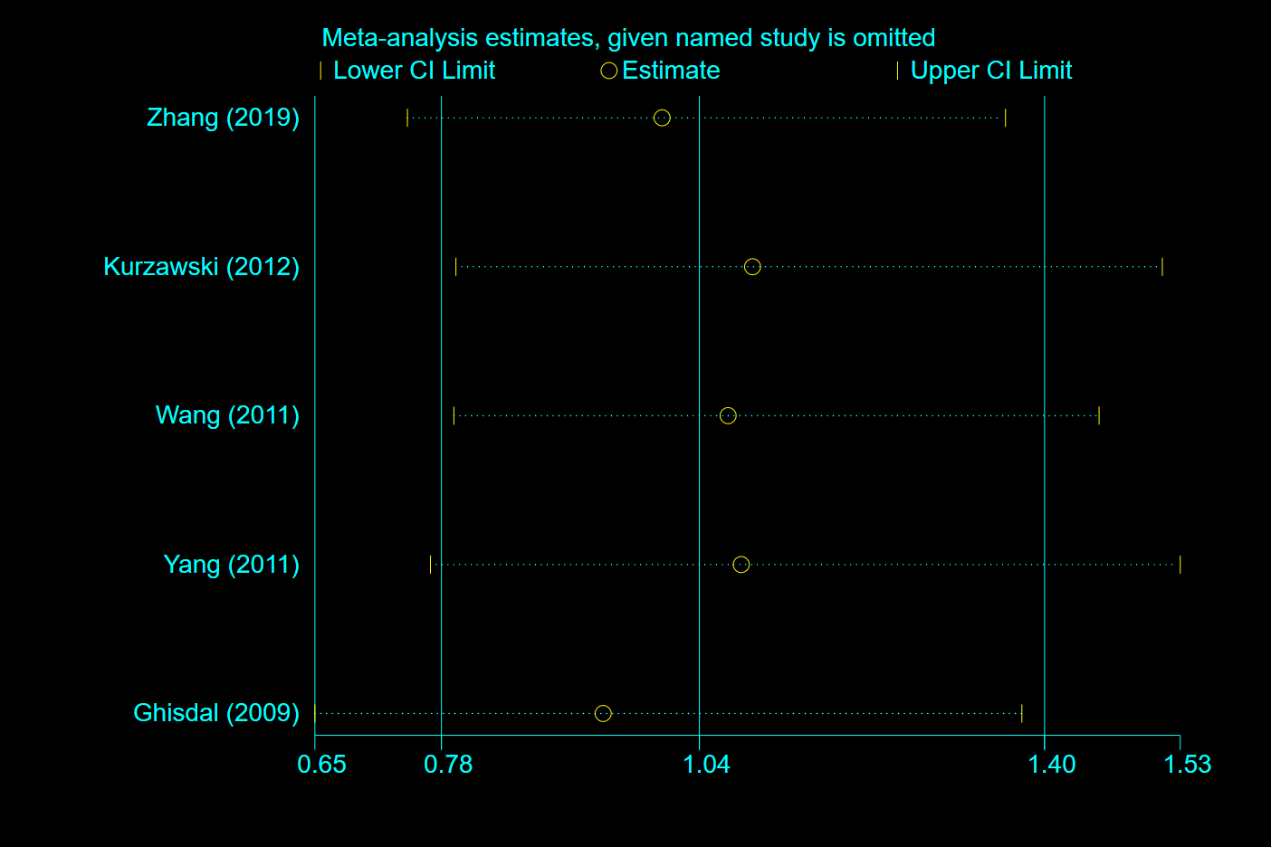
**

**PPARγ(rs1801282)-Heterozygote model**

**
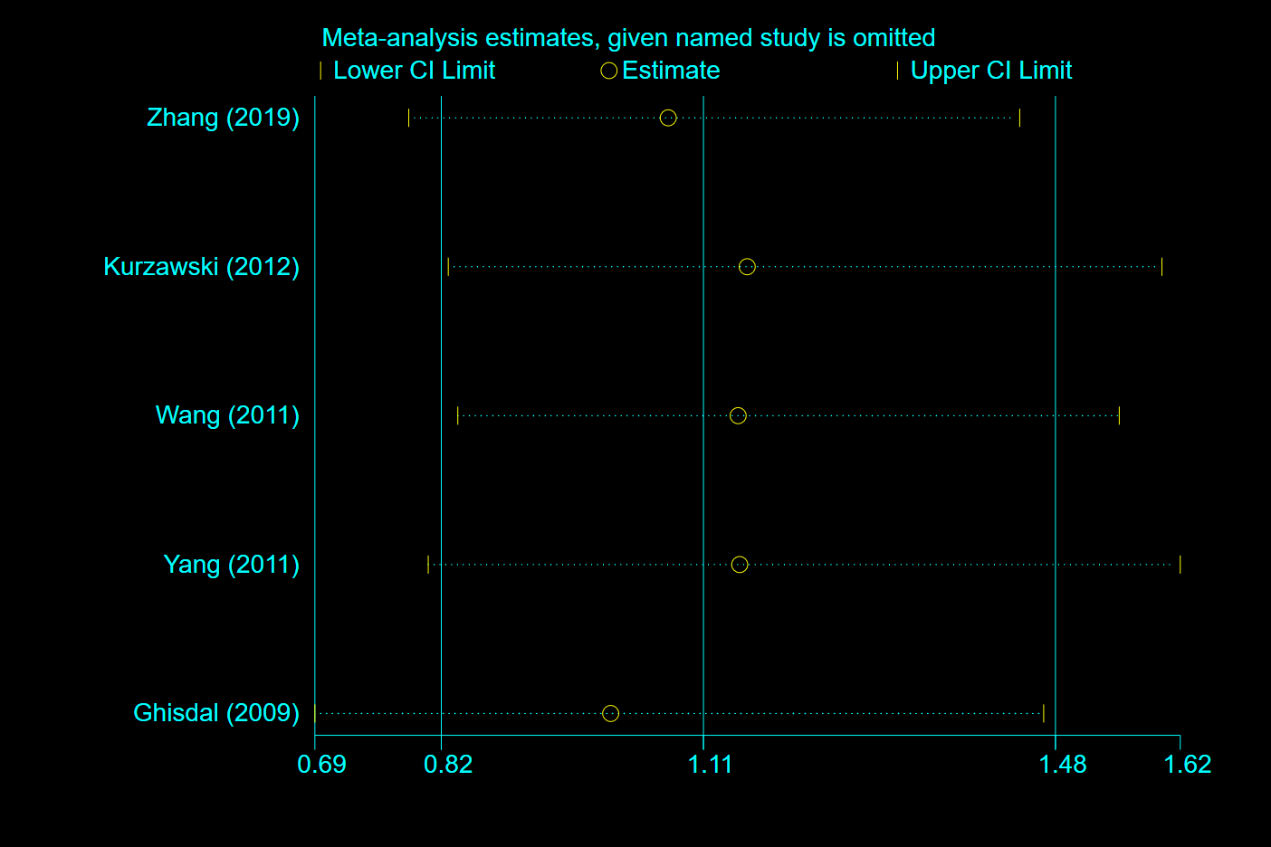
**

**PPARγ(rs1801282)-Homozygote model**

**
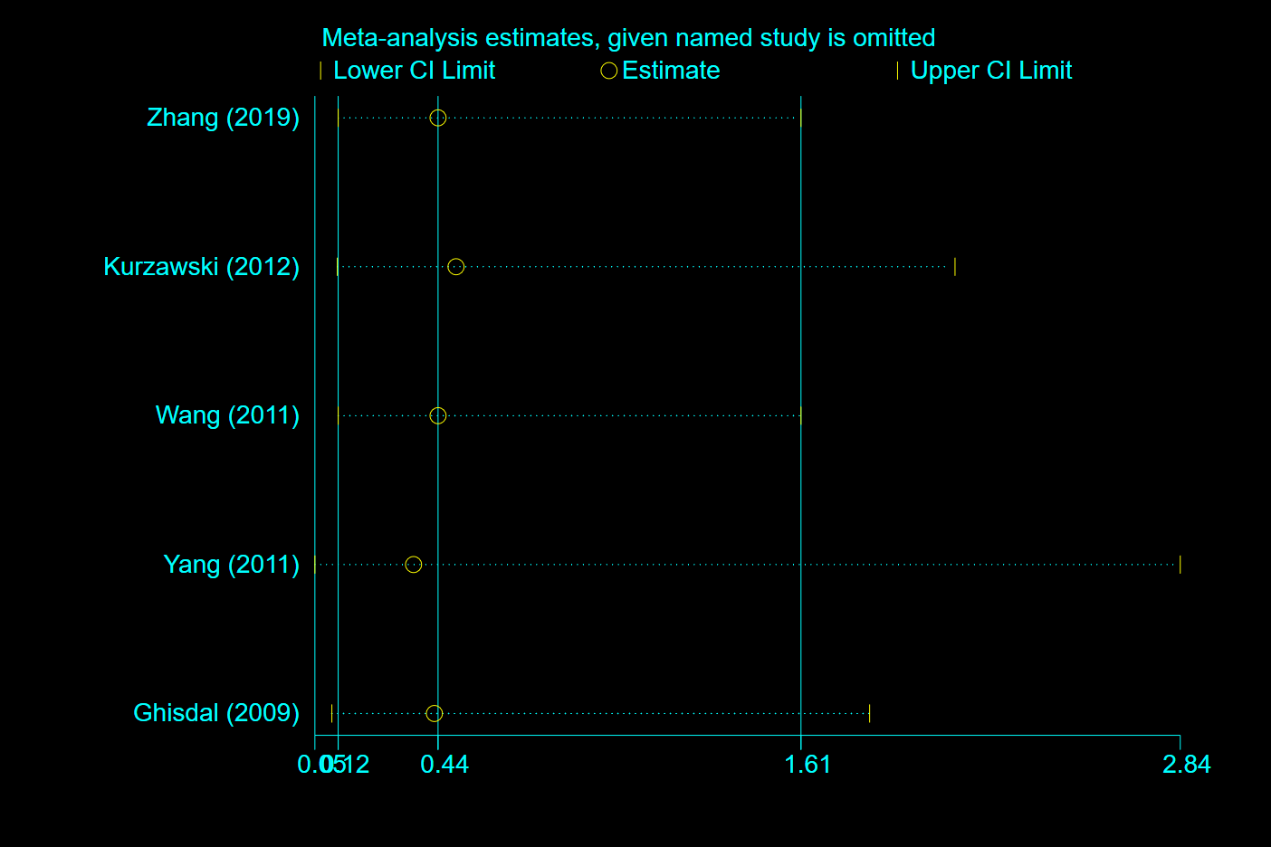
**

**PPARγ(rs1801282)-Recessive model**

**
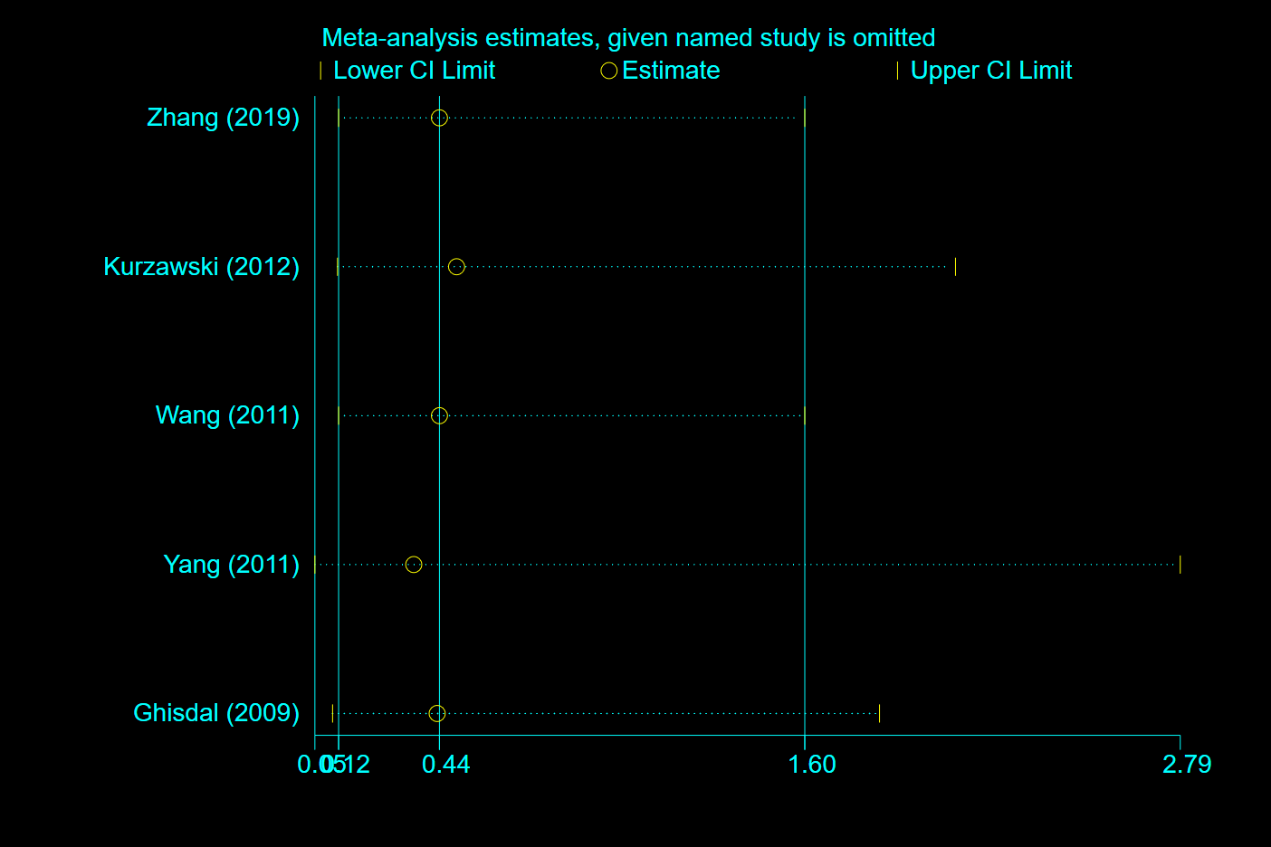
**
