# Supplementary material for: Association between Genetic Polymorphisms and Risk of Kidney Posttransplant Diabetes Mellitus: A Systematic Review and Meta-Analysis
Source: Int J Clin Pract. 2022 Mar 8;2022:7140024. doi: 10.1155/2022/7140024 (PMC9159121; doi:10.1155/2022/7140024)
Supplement: Supplementary Materials — The supplementary materials contain supplementary figures and tables and PRISMA checklist. [file 7140024.f1.zip › 7140024.f1/Supplementary Table 1 (1).pdf]

Base Information																outcomes	
Study name	Study ID	N(Total)	Country/Ethnicity	Design	Genotyping methods	Types of calcineurin inhibitors	Diagnostic criteria of cases	Time of PTDM diagnosis	Source of controls	Sample size		Age at transplantation (mean ± SD), y	Gender female (%)	Blood glucose, mmol/L(prior to transplantation)	Blood glucose, mmol/L(Follow up)	genetype	MAF (%)
										PTDM	/Non-	PTDM /Non-PTDM	PTDM /Non-PTDM	PTDM /Non-PTDM	PTDM /Non-PTDM		
Serum magnesium, hepatocyte nuclear factor 1β genotype and post-transplant diabetes mellitus: a prospective study	Van der Burgh,2020	167	Netherlands/	Embedded within an investigator-initiated,prospective,randomized-controlled,parallel group,open-label,single centre clinical trial	TaqMan Assay reagents for allelic discrimination	Tacrolimus	American Diabetes Association as a fasting blood glucose>7.0mmol/L, or a non-fasting blood glucose >11.1 mmol/L	1 year after transplantation	Hospital	29	138	60±7/51±15	34.5/41.3	5.7±0.35/5.1± 0.28	6.6±0.73/5.5±0.33	HNF1b(rs752010 G > A, rs4430796 A > G and rs7501939 C > T)	
Clinical and genetic risk factors for new-onset diabetes mellitus after transplantation (NODAT) in major transplant centres in Malaysia	Guad,2020	168	Malaysia/Malay, Chinese, Indian	Cohort	Sequenom Mass Array platform (Sequenom, USA).	Cyclosporine/Tacrolimus/Both	American Diabetes Association FBG ⩾126 mg/dl (7.0 mmol/l) and not on oral hypoglycaemic agents or insulin at any point of follow up period within one-year of renal transplant	Within 1 year of transplantation	Hospital	29	139	39.3 ± 13.4/33.9 ± 11.8	44.8/40	/	6.5 ± 1.8/5.2 ± 0.7	IL-7R(rs1494558) and mannose binding leptin-2, MBL(rs2232365)	
Variability in the leptin receptor gene and other risk factors for post-transplant diabetes mellitus in renal transplant recipients	Mota-Zamorano,2019	315	Spain/Caucasian	Cohort	RT-PCR	Cyclosporine/Tacrolimus	American Diabetes Association criteria and were defined as two fasting plasma glucose values 6.99mmol/l or symp-toms of diabetes plus casual plasma glucose concentra-tions 11.10mmol/l throughout the first year	1 year after transplantation	Hospital	57	258	/	/	/	/	LEPR: Lys109Arg (rs1137100), Gln223Arg (rs1137101) and Lys656Asn (rs1805094)	
Validation of Identified Susceptible Gene Variants for New-Onset Diabetes in Renal Transplant Recipients	Hwang,2019	1102	Korean/	Prospective, multicenter, nationwide cohort study	TaqMan-based QuantStudio OpenArray	Tacrolimus/Steroid	American Diabetes Association,NODAT was diagnosed when fasting blood sugar was higher than 126 mg/dL six months after transplantation, or when insulin or oral hypoglycemic agents were required for treatment	Within 1 year of transplantation	Hospital	254	848	52.2 ± 10.4/45.1 ± 12.0	40.2/47.5	/	/	CDKAL1(rs10946398),KCNQ1(rs2237892),ATP5F1P6(rs10484821), DNAJC16(rs7533125),CELA2B(rs2861484),CASP9(rs2020902), NOX4(rs1836882),INPP5A(rs4394754),IL7R(rs2172749),IL17R(rs4819554),IL17RB(rs1025689,rs1043261),PLXDC1(rs72823322)	
Genetic risk factors for post-transplantation diabetes mellitus in Chinese Han renal allograft recipients treated with tacrolimus	Zhang,2019	129	China/Chinese,Han	Cohort	PCR-RFLP	Triple-therapy/Tacrolimus,mycophenolate mofetil,Steroid	American Diabetes Association,PTDM was defined as hemoglobin A1c continuously >6.5 mg/dL, fasting plasma glucose of >126 mg/dL (7.0 nmol/L), or those requiring insulin and/or oral hypoglycemic agents for >3 months	6 months after transplantation	Hospital	17	112	49.35± 9.06/46.56± 9.91	29.4/23.2	/	/	CYP3A5(rs776741, rs776746, rs15524), CYP24A1 (rs2296241), and PPARG (rs1801282)	
Association of the PCK2 Gene Polymorphism With New-onset Glucose Intolerance in Japanese Kidney Transplant Recipients	Yokoyama,2018	38	Japan/Japanese	Cohort	TaqMan SNP Genotyping Assay	Cyclosporine/Tacrolimus	Patients withplasma glucose level >140 mg/dL at 120 minutes in the 75-g OGTT at 1 year after KTx	1 year after transplantation	Hospital	11	27	37.3 ± 9.0/44.6 ± 15.0	27.2/44.4	/	/	SLC2A2(rs1499821,rs5398),PCK2(rs4982856),IGF2BP2(rs4402960),CDKN2A/B(rs10811661),HHEX(rs1111875),SLC30A8(rs13266634) and CDKAL1(rs7756992)	
CYP3A4 and GCK genetic polymorphisms are the risk factors of tacrolimus-induced new-onset diabetes after transplantation in renal transplant recipients	Shi,2018	169	China/Chinese,Han	Case-Control	PCR	Tacrolimus	Patients were diagnosed as NODAT by fasting plasma glucose ⩾ 7.0 mmol/L, 2 h post-load glucose ⩾ 11.1 mmol/L during oral glucose tolerance test or required blood sugar control with insulin and/or oral hypoglycemic drugs for more than 3 months after transplantation.	3 months after transplantation	Hospital	57	112	43.1 ± 9.0/38.6 ± 11.8	/	/	7.43 ± 0.86/4.27 ± 0.72	CYP3A4 *18B,CYP3A5*3,ABCC8 T-3C,and GCK G-30A	
Evaluation of Glutathione Peroxidase and KCNJ11 Gene Polymorphisms in Patients with New Onset Diabetes Mellitus After Renal Transplantation	Yalin,2017	118	Turkey	Monocenter case-control	PCR-RFLP	CSA + AZA + PRED/CSA + MMF + PRED/TAC + MMF + PRED	American Diabetes Associa-tion guidelines: fasting plasma glucose (FPG) ⩾ 126 mg/dL or 2hPG ⩾ 200 mg/dL during an oral glucose tolerance test (OGTT) or HbA1c ⩾ 6.5 % or random PG ⩾ 200 mg/dL in the presence of classic symptoms of hyperglycaemia	/	Hospital	58	60	47.2 ± 11.0/38.5 ± 10.1	31/36.7	/	/	GPX1(rs1050450) and KCNJ11(rs5219)	
KCNJ11 and KCNQ1 Gene Polymorphisms Are Not Associated with Post-Transplant Diabetes Mellitus in Kidney Allograft Recipients Treated with Tacrolimus	Dabrowska-Zamojcin, 2017	201	Poland	Cohort	RT-PCR	Standard immunosuppression consisted of tacrolimus, mycophenolate mofetil and	The diagnosis of diabetes was made when one of the following plasma glucose parameters was ex-ceeded: fasting plasma glucose 5.5 mmol/l and 2 h plas-ma glucose 7.8 mmol/l.	8.6 months after transplantation	Hospital	35	166	/	/	/	/	KCNJ11(rs5219),KCNQ1(rs151290),KCNQ1(rs2237892)	
New-onset diabetes after transplant: Incidence, risk factors and outcome	Alagbe,2017	111	South Africa	Cohort	PCR	Cyclosporine/Tacrolimus	Fasting plasma glucose (FPG) >7 mmol/L or random plasma glucose >11.1 mmol/L	Tacrolimus treatment arm 12 months/36 months for those treated with cyclosporine	Hospital	20	91	44/37	37.4/50	/	8.1/5.8	TCF7L2(rs11196205, rs12255372 and rs7903146) and HNF1β (rs1800575, rs121918671 and rs121918672)	
Matrix Metalloproteinase Gene Polymorphisms and New-Onset Diabetes After Kidney Transplantation in Korean Renal Transplant Subjects	Ong,2017	309	Korea	Cohort	PCR	Tacrolimus/other	NODAT was defined when HbA1c was >6.5%, fasting blood sugar levels were >7.0 mmol/L (126 mg/dL)		Hospital	52	257	45.11± 9.90/38.26± 11.17	46.4/39.2	/	/	MMP-1(rs1144393,rs475007,rs494379,rs470558),MMP-2(rs17859821,rs1132896,rs1053605,rs243849),MMP-3(rs679620,rs602128,rs520540)	
Significant Association between Toll-Like Receptor Gene Polymorphisms and Posttransplantation Diabetes Mellitus	Kim,2016	305	Korea	Cohort	PCR	Cyclosporine/Tacrolimus/Other	American Diabetes Association guidelines	3 months after transplantation	Hospital	51	254	45.56±1.28 /38.28±0.71	47.1/39.4	/	/	TLR2(rs3804099,0rs3804100),TLR4(rs1927914),TLR6 (rs3775073,rs3821985,rs1039559)	
STAT4 gene polymorphism in patients after renal allograft transplantation	Dabrowska-Zamojcin, 2016	169	Poland	Cohort	RT-PCR	Triple-drug therapy, including calcineurin in-hibitors (cyclosporine A in 75% and tacrolimus in 24% of recipients), azathioprine (55%) or mycophenolate mofetil (37%), and steroids (91%)	Patients with haemoglobin A1c continuously over 6.5%, fasting blood glucose ≥ 7.0 mmol/l, or requiring treatment with oral hypoglycaemic agents or insulin for more than three months after transplantation were diagnosed as hav-ing post-transplant diabetes mellitus (PTDM)	3 months after transplantation	Hospital	23	146	/	/	/	/	STAT4(rs7574865)	
Adiponectin and leptin gene polymorphisms in patients with post-transplant diabetes mellitus	Romanowski,2015a	323	Poland/Caucasian	Cohort	RT-PCR	Tacrolimus/cyclosporine	Patients with hemoglobin A1c contin-uously over 6.5%, fasting blood glucose ≥ 7.0 mmol/l or requiring treatment with oral hypoglycemic agents or insulin for more than 3 months after transplanta-tion were diagnosed as having PTDM	3 months after transplantation	Hospital	43	272	/	/	/	/	ADIPOQ(rs266729,rs1501299),LEP(rs2167270)	

Interleukin-17 gene polymorphisms in patients with post-transplant diabetes mellitus	Romanowski,2015b	167	Poland/Caucasian	Cohort	RT-PCR	Standard im-munosuppression consisted of tacrolimus, my-cophenolate mofetil, and steroids	Patients with haemoglobin A1c continuously over 6.5%, fasting blood glucose ≥ 7.0 mmol/l, or requiring treatment with oral hypoglycaemic agents or insulin for more than 3 months after transplantation were diagnosed as having PT-DM	3 months after transplantation	Hospital	23	146	/	/	/	/	IL17A(rs2275913) and IL17F(rs11465553,rs2397084,rs763780)
Validation of the association of TCF7L2 and SLC30A8 gene polymorphisms with post-transplant diabetes mellitus in Asian Indian population	Khan,2015	142	India	Cohort	PCR-RFLP	CsA/Tacrolimus	American Diabetes Association	3 months after transplantation	Hospital	42	98	39.57±11.8/39.48±10.59	28.6/23.5	/	/	TCF7L2(rs7903146 and rs13266634) and SLC30A8(rs13266634)
Tacrolimus decreases insulin sensitivity without reducing fasting insulin concentration: a 2-year follow-up study in kidney transplant recipients	Chen,2015	293	China/Chinese	Cohort	PCR	Tacrolimus	World Health Organization,All patients who showed random plasma glucose concentration 11.1 mmol/L or fasting plasma glucose (FPG) 7.0 mmol/L or 2-h plasma glucose level 11.1 mmol/L following oral glucose tolerance test (OGTT) on two consecutive days were confirmed to be diabetic	1 months after transplantation	Hospital	78	80	40.4±9.4 /38.7±8.2	25.6/26.3	4.56 ± 0.58/4.36 ± 0.60	7.4 ± 3.14/4.88 ± 0.67	IRS-2 Gly1057Asp(G1057A),IRS-1 Gly972Arg(G972C)
Impact of PPARA and POR polymorphisms on tacrolimus pharmacokinetics and new-onset diabetes in kidney transplant recipients	Kurzawski,2014	241	Poland/White	Cohort	RT-PCR	Tacrolimus	Patients with fasting plasma glucose over 126 mg/dl (7.0 nmol/l) or requiring insulin and/or oral hypoglycemic agents for more than 3 months were diagnosed with NODAT.	1 year after transplantation	Hospital	48	176	/	/	/	/	POR(rs1057868 *28),PPARA(rs4253728,rs4823613)
The incidence of posttransplantation diabetes mellitus during follow-up in kidney transplant recipients and relationship to Fok1 vitamin D receptor polymorphism	Yao,2013	105	China/Chinese	Cohort	PCR-RFLP	Mycophenolate mofetil (MMF) and corticosteroids	American Diabetes Association (ADA) guidelines	6 months after transplantation	Hospital	16	89	47.81±15.54/36.62±11.43	37.5/34.8	/	/	VDR Fok1
Association between 276G/T adiponectin gene polymorphism and new-onset diabetes after kidney transplantation	Nicoletto, 2013	270	Brazil/Caucasian	Cohort	RT-PCR	Cyclosporine/Tacrolimus	American Diabetes Association criteria,Diagnosis of NODAT was made at the second FPG of 126 mg/dL or more.	1 year after transplantation	Hospital	83	187	48.1±11.0/39.8 ±11.9	39.6/39.8	5.33±0.29/4.94±0.19	/	Adiponectin-276G/T,CCL5(rs2280789,rs3817655)
Angiotensinogen polymorphisms and post-transplantation diabetes mellitus in Korean renal transplant subjects	Lee,2013	302	Korea	Cohort	PCR	Tacrolimus /Other	American Diabetes Association guidelines,asting blood glucose levels ≥126 mg/dl (7.0 mmol/l); or symptoms of diabetes mellitus plus plasma glucose concentrations ≥200 mg/dl (11.1 mmol/l) at any time of day; or 2-h post-load glucose ≥200 mg/dl (11.1 mmol/l) during an oral glucose tolerance test; or insulin and/or oral hypoglycemic agents that were required for more than three months	3 months after transplantation	Hospital	49	253	45.18±9.39/38.1±11.21	46.9/38.7	/	/	ACE(rs4291),AGT(rs699,rs4762)
Single-nucleotide polymorphisms in P450 oxidoreductase and peroxisome proliferator-activated receptor-α are associated with the development of new-onset diabetes after transplantation in kidney transplant recipients treated with tacrolimus	Elens,2013	101	Belgium	Cohort	RT-PCR	Tacrolimus	/	at any time	Hospital	9	76	/	/	/	/	PPARa(rs4253728),POR(rs1057868 *28)
Gene polymorphisms are associated with posttransplantation diabetes mellitus among Taiwanese renal transplant recipients	Weng,2012	278	China/Taiwan	Cohort	PCR-RFLP	Cyclosporine/Tacrolimus	All participants with PTDM were diagnosed according to the 2003 international consensus guidelines for new-onset diabetes after transplantation	/	Hospital	27	251	47.6±9.8/41.7±11.5	44.6/22.2	/	/	PAI-1-675(4G/5G),IL-1-511,IL-6-174,GLUT1,MTHFR-677
Analysis of common type 2 diabetes mellitus genetic risk factors in new-onset diabetes after transplantation in kidney transplant patients medicated with tacrolimus	Kurzawski,2012	235	Poland/Caucasian	Cohort	RT-PCR	Tacrolimus	Patients with hemoglobin A1c continuously >6.5 mg/dL, fasting plasma glucose of >126 mg/dL (7.0 nmol/L), or those requiring insulin and/or oral hypoglycemic agents for >3 months were diagnosed as having NODAT	1 year after transplantation	Hospital	67	168	47.7±10.6/43.2 ±13.0	45.5/46.4	/	/	IGF2BP2(rs4402960,rs1470579),HHEX(rs1111875),CDKN2A/B(rs10811661),SLC30A8 (rs13266634),PPARG(rs1801282), KCNJ11 (rs5215)
Association of genetic polymorphisms of interleukins with new-onset diabetes after transplantation in renal transplantation	Kim,2012	306	Korea	Cohort	PCR	Tacrolimus /Other	NODAT was diagnosed when the FPG concentration was over 126 mg/dL, HbA1c was more than 6.5%, or insulin and oral hypoglycemic agents were required for over 3 months	3 months after transplantation	Hospital	53	253	44.91±1.33/38.34±0.71	47.2/39.5	4.9±0.03/4.99±0.09	/	IL-1B (rs3136558), IL-2 (rs2069762), IL-4 (rs2243250, rs2070874), IL-7R (rs1494558, rs2172749), IL-17RE (rs1124053), IL-17R (rs2229151, rs4819554), and IL-17RB (rs1043261, rs1025689)
Variants of the adiponectin and adiponectin receptor-1 genes and posttransplantation diabetes mellitus in renal allograft recipients	Kang,2012	575	Korea	Cohort	PCR	Cyclosporin A/tacrolimus	The International Consensus Guidelines for the diagnosis and management of PTDM	1 year after transplantation	Hospital	154	421	42.3±9.2/37.3±9.4	37.7/35.6	5.09±0.69/5.21±0.77	7.08±2.6/5.39±0.81	ADIPOQ(rs266729,rs822395,rs822396,rs2241766, and rs1501299 and ADIPOR1 rs2232853, rs12733285, and rs1342387)
Adiponectin gene polymorphisms are associated with posttransplantation diabetes mellitus in Chinese renal allograft recipients	Yu,2011	398	China/Chinese	Cohort	PCR	Cyclosporine (CsA) or tacrolimus, mycophenolate or azathioprine, and steroid.	PTDM was defined according to the American Diabetes Associa-tion criteria.16 All patients were confirmed to show fasting plasma glucose level (FPG) 7 mmol/L on at least two occasions or to require antidiabetic treatment (oral	Any time in two years	Hospital	97	301	45.55±10.78/40.26±11.47	19.6/33.9	5.19±0.79/5.14±0.67	6.56±1.64/5.47±0.76	Adiponectin(-276,-45)
Genetic and clinical risk factors of new-onset diabetes after transplantation in Hispanic kidney transplant recipients.	Yang,2011	303	USA	Cohort	RT-PCR	Cyclosporine or tacrolimus), mycophenolic acid derivatives, sirolimus, and prednisone depending on the patients' clinical condition	Based on definition by the American Diabetic Association, the patients who had no evidence of diabetes before transplantation but had at least two occasions of fasting plasma glucose (FPG) level more than 126 mg/dL 1 month or later after transplantation were defined as NODAT			133	170	44.30±13.79/41.01±13.11	43.6/43.5	/	/	TCF7L2(rs7903146,rs12255372),HNF4A(rs2144908,rs1800961,rs1884614), HNF1A(rs1800574,rs1169288),KCNJ11(rs5219),SUR1(rs1799854,rs1801261), ENPP1(rs1044498),PPARG(rs1801282),PPARGC1(rs8192678),and IRS1(rs1801278)
Increased body mass index but not common vitamin D receptor, peroxisome proliferator-activated receptor γ, or cytokine polymorphisms confers predisposition to posttransplant diabetes	Wang,2011	123	UAS/White,African American,Hispanic,Asian	Case-Control	PCR	Tacrolimus and mycophenolate mofetil	Posttransplant diabetes mellitus was defined as fasting glucose $126 mg/dL (to convert to millimoles per liter, multiply by 0.0555)	3 months after transplantation	Hospital	51	72	49.02±13.04/47.22±12.83	45.1/37.5	/	/	VDR (taqI t/T，rs731236),IFNg (+874T/A,rs2430561), PPARg (P12A,rs1801282), TGFb1 (2869T/C, rs1800470 (formerly rs1982073)), and TNF (2308G/A,rs1800629)

Glutathione S-transferase gene polymorphisms are not major risks for susceptibility to posttransplantation diabetes mellitus in Taiwan renal transplant recipients	Tsai,2011	283	China/Taiwan	Cohort	PCR-RFLP	Tacrolimus	American Diabetes Association guidelines. These criteria were: fasting blood glucose Z126mg/dl (7.0 mmol/l); or symptoms of diabetes plus plasma glucose concentration Z200mg/dl (11.1mmol/l) at any time of day; or 2hr postload glucose Z200mg/dl (11.1 mmol/l) during an oral glucose tolerance test	19.27±26.3 months after transplantation	Hospital	85	198	54.9±9.36/50.6±11	45.9/50	/	/	GSTA1, GSTP1, and GSTM1 genotypes
KCNQ1 gene variants and risk of new-onset diabetes in tacrolimus-treated renal-transplanted patients	Tavira,2011	405	Spain/Caucasian	Cohort	PCR-RFLP	Standard triple immunosuppressive therapy with Tac, mycophenolate mofetil (MMF), and	Defined as a fasting plasma glucose >125 mg/dL, 7.0 mM, after three consecutive measurements	12 months after transplantation	Hospital	145	260	49±11/44±13	40/38	/	6.8 ± 1.96/5.07 ± 0.66	KCNQ1(rs2237895 (A/C),rs2237892 (C/T) and rs8234)
Angiotensinogen t235 and angiotensin-converting enzyme insertion/deletion polymorphisms associated with the development of posttransplantation diabetes mellitus in renal allograft recipients	Ö zdemir,2011	50	Turkey	Cohort	PCR	Standard triple immunosuppressive therapy with Tac, mycophenolate mofetil (MMF), and prednisone	Diabetes mellitus was diagnosed in accordance with American Diabetes Association/World Health Organization guidelines as symptoms of diabetes plus any time plasma glucose concentrations ≥200 mg/dL, or finding of ≥2 consecutive fasting glycemia ¬126 mg/dL or 2-hour plasma glucose ≥200 mg/dL during oral glucose tolerance test and the need for insulin or oral antidiabetic therapy	12 months after transplantation	Hospital	23	27	37.9±10.5/38.3±10.9	33.3/35	/	/	ACE insertion/deletion (I/D) and AGT M235T
Association of transcription factor 7-like 2 (TCF7L2) gene polymorphism with posttransplant diabetes mellitus in kidney transplant patients medicated with tacrolimus	Kurzawski,2011	234	Poland	Cohort	RT-PCR	Tacrolimus, mycophenolate mofetil and steroids	Patients with hemoglobin A1c (HgA1c) levels continuously over 6.5 mg/dl, fasting plasma glucose (FPG) levels over 126 mg/dl, or who required insulin and/or oral hypoglycemic agents for more than 3 months were diagnosed as having PTDM	12 months after transplantation	Hospital	66	168	47.7±10.6/43.2±13.0	45.5/46.4	/	/	TCF7L2(rs12255372 and rs7903146)
Increased body mass index after kidney transplantation in activating transcription factor 6 single polymorphism gene carriers	Fougeray, 2011	269	France/Caucasians,Black, Asiatics, Other/unknown	Cohort	PCR	Tacrolimus, mycophenolate mofetil	NODAT was defined in a previously nondiabetic patient as the occurrence of a fasting glucose level ¬7 mmol/L or any nonfasting glycemia ¬11 mmol/L measured at baseline or at days 14, 30, 60, or 90.	3 months after transplantation	Hospital	21	248	/	/	/	/	ATF6(rs10918215, rs7514053, rs1058405, rs4479731, rs2340721, and rs13401)
Plasminogen activator inhibitor-1 5G/5G genotype is a protecting factor preventing posttransplant diabetes mellitus	Chang,2011	376	China/Taiwan	Cohort	PCR-RFLP	Cyclosporine or tacrolimus, mycophenolate mofetil (MMF) or mycophenolic acid with or without prednisolone	PTDM was diagnosed according to the American Diabetes Association guidelines . These criteria were: fasting blood glucose⩾126 mg/dl (7.0 mmol/l); or symptoms of diabetes plus plasma glucose concentration⩾200 mg/dl (11.1 mmol/l) at any time of day; or 2-h postload glucose⩾200 mg/dl (11.1 mmol/l) during an oral glucose tolerance test	Any time in follow up	Hospital	81	259	55.3±10.0/52.6±11.3	43.2/48.4	/	/	PAI-1 −675 4G/5G
Association of calpain-10 gene polymorphism and posttransplant diabetes mellitus in kidney transplant patients medicated with tacrolimus	Kurzawski,2010	214	Poland	Cohort	PCR	Tacrolimus, mycophenolate mofetil and steroids	Patients with hemoglobin A1c continuously over 6.5mg per 100 ml, fasting plasma glucose over 126mg per 100ml or requiring insulin and/or oral hypoglycemic agents for more than 3 months at 1 year after transplantation were diagnosed as having PTDM	12 months after transplantation	Hospital	56	158	47.3±9.9/43.0±13.2	51.8/52.5	/	/	CAPN10(rs3792267:G4A, rs3842570 ins/del and rs5030952:C4T)
Tumor necrosis factor alpha promoter polymorphism in posttransplantation diabetes mellitus of renal transplant recipients	Kao,2010	314	China/Taiwan	Cohort	PCR-RFLP	CsA/FK506	(HbA1c) ＞6.5 mg/dL on sequential blood samples or requiring insulin after transplantation who were diagnosed with PTDM	Any time in follow up	Hospital	73	241	49.4±9.37/47±10.85	42.5/47.3	/	/	TNF-α（G-238A and G-308A）
Significant associations between CCL5 gene polymorphisms and post-transplantational diabetes mellitus in Korean renal allograft recipients	Jeong,2010	311	Korea	Cohort	PCR	Tacrolimus/others	PTDM was diagnosed when hemoglobin A1c was continuously over 6.5%, fasting plasma glucose concentration was over 126 mg/dl, or insulin and/or oral hypoglycemic agents were required for over 3 months.	3 months after transplantation	Hospital	56	255	45.11±9.90/38.26±11.17	46.4/39.2	/	/	CCL5(s2107538 in promoter –403, rs2280789 in intron_1, and rs3817655 in intron_2)
Polymorphisms of superoxide dismutase, glutathione peroxidase and catalase genes in patients with post-transplant diabetes mellitus	Dutkiewicz,2010	159	Poland/Caucasian	Cohort	PCR-RFLP	Standard immunosuppression consisted of tacrolimus, mycophenolate mofetil (MMF),	Patients with hemoglobin A1c continuouslyO6.5%, fasting blood glucose $7.0 mmol/l, or requiring treatment with oral hypoglycemic agents or insulin continued for O3 months after transplantation were diagnosed as having PTDM	3 months after transplantation	Hospital	21	138	46.8±8.8/42.0±13.6	33.3/43.5	/	/	CAT(-262C/T),SOD1(-239þ34A/C) SOD2(47C/T) and GPX1 gene (-C599T,rs1050450)
Association of common type 2 diabetes risk gene variants and posttransplantation diabetes mellitus in renal allograft recipients in Korea	Kang,2009	589	Korea	Cohort	PCR	Calcineurin inhibitors and glucocorticoids	PTDM was diagnosed 1 year posttransplantation according to the International Consensus Guidelines for the diagnosis and management ofPTDM	12 months after transplantation	Hospital	145	444	42.6±9.1/37.4±9.3	35.2/34.7	5.27±0.84/5.15±0.7	7.18±3.06/5.40±0.83	TCF7L2(rs7903146), SLC30A8(rs13266634), HHEX (rs1111875, rs7923837, and rs5015480), CDKAL1(rs10946398,) CDKN2A/B(rs10811661), IGF2BP2(rs4402960), FTO (rs8050136), WFS1(rs734312), JAZF1(rs864745), CDC123/CAMK1D(rs127797900, TSPAN8(rs7961581), THADA(rs7578597), ADAMTS9(rs4607103), NOTCH2(rs1092391), and KCNQ1(rs2237892)
TCF7L2 polymorphism associates with new-onset diabetes after transplantation	Ghisdal,2009	1076	France	Cohort	RT-PCR	Cyclosporin A/tacrolimus/mTOR inhibitor	NODAT was defined by either the 2003 diagnostic criteria of the American Diabetes Association36 (fasting plasma glucose [FPG] ≥126 mg/dl [7.0 mmol/L] on at least two occasions) at 3 or 6 mo and/or de novo prescription ofhypoglyemic therapy within 6mo after transplantation	6 months after transplantation	Hospital	118	958	52.8/46.7	42.4/37.1	/	/	TCF7L2(rs7903146), FTO(rs8050136), CDKAL1(rs7754840), KCNJ11(rs5215), HHEX-IDE(rs11118750),SLC30A8(rs13266634), CDKN2A-CDKN2B(rs10811661), IGF2BP2(rs4402960),HNF1B(rs757210),WFS1(rs10010131), and PPARγ(rs1801282)
A polymorphism in the zinc transporter gene SLC30A8 confers resistance against posttransplantation diabetes mellitus in renal allograft recipients	Kang,2008a	624	Korea	Cohort	PCR	Cyclosporine/Tacrolimus	PTDM was diagnosed according to American Diabetes Association criteria (26) at the 3rd month posttransplantation	3 months after transplantation	Hospital	174	450	42.1±8.99/35.42±9.43	35.1/35.6	5.31±1.35/5.16±1.44	6.93±2.85/5.41±0.85	SLC30A8(rs13266634)
A variant of the transcription factor 7-like 2 (TCF7L2) gene and the risk of posttransplantation diabetes mellitus in renal allograft recipients	Kang,2008b	511	Korea	Cohort	RT-PCR	Cyclosporine A or tacrolimus) and glucocorticoids	PTDM was diagnosed according to American Diabetes Association criteria (21) after the third posttransplantation month	3 months after transplantation	Hospital	119	391	41.10±9.33/35.64±10.8	34.5/36.5	5.31±1.54/5.11±1.37	6.85±3.02/5.35±0.94	TCF7L2(rs11196205, rs4506565, rs12243326, rs7903146, rs12255372, and rs7901695)
