# Supplementary material for: Association between Genetic Polymorphisms and Risk of Kidney Posttransplant Diabetes Mellitus: A Systematic Review and Meta-Analysis
Source: Int J Clin Pract. 2022 Mar 8;2022:7140024. doi: 10.1155/2022/7140024 (PMC9159121; doi:10.1155/2022/7140024)
Supplement: Supplementary Materials — The supplementary materials contain supplementary figures and tables and PRISMA checklist. [file 7140024.f1.zip › 7140024.f1/Supplementary Table 2 (1).pdf]

		Case Group		Control Group			Case Group		Control Group	
		Expose	Unexposed	Expose	Unexposed		Expose	Unexposed	Expose	Unexposed
**TCF7L2(rs7903146)**	Allele model	E1	NOE1	C1	NOC1	Dominant model				
	Alagbe,2017	11	17	30	76	7	8	6	26	27
	Khan,2015	40	44	49	147	Khan,2015	29	13	41	57
	Yang,2011	50	216	72	268	Yang,2011	45	88	65	105
	Kurzawski,2011	41	91	87	249	Kurzawski, 2011	34	32	77	91
	Kang,2009	16	274	23	865	Kang,2009	16	129	23	421
	Ghisdal,2009	86	150	541	1373	09	71	47	458	499
	Kang,2008b	12	226	17	767	b	12	107	17	375
**TCF7L2(rs12255372)**										
	Alagbe,2017	9	21	23	93	7	8	7	20	38
	Yang,2011	51	215	67	273	Yang,2011	44	89	60	110
	Kurzawski,2011	40	92	84	252	Kurzawski, 2011	34	32	73	95
**SLC30A8(rs13266634)**										
	Yokoyama,2018	8	14	19	35	Yokoyama, 2018	6	5	16	11
	Khan,2015	32	52	46	150	Khan,2015	26	16	39	59
	Kurzawski,2012	96	38	111	225	Kurzawski, 2012	63	4	95	73
	Kang,2009	102	188	401	487	Kang,2009	84	61	314	130
	Ghisdal,2009	66	170	566	1348	09	57	61	483	474
	Kang,2008a	126	222	402	498	a	104	70	313	137
**KCNQ1(rs2237892)**										
	Hwang,2019	151	357	667	1029	9	126	128	531	317
	Dabrowska-Zamojcin,2017	5	65	17	315	- Zamojcin,2017	4	31	17	149
	Tavira,2011	12	278	25	495	1	9	136	23	237
	Kang,2009	86	204	339	549	Kang,2009	69	76	273	171
**PPARγ(rs1801282)**										
	Zhang,2019	4	30	15	209	9	4	13	15	97
	Kurzawski,2012	15	119	47	289	Kurzawski, 2012	15	52	43	125
	Wang,2011	6	96	11	133	1	6	45	11	61
	Yang,2011	32	234	45	295	Yang,2011	30	103	40	130
	Ghisdal,2009	26	210	188	1722	09	26	92	180	775
**CDKN2A/B(rs10811661)**										
	Yokoyama,2018	11	11	29	25	Yokoyama, 2018	9	2	23	4
	Kurzawski,2012	120	14	287	49	Kurzawski, 2012	67	0	166	2
	Kang,2009	182	108	496	392	Kang,2009	129	16	361	83
	Ghisdal,2009	180	54	1529	371	09	111	6	899	51
**HHEX(rs1111875)**										
	Yokoyama,2018	7	15	26	28	Yokoyama, 2018	5	6	19	8
	Kurzawski,2012	56	78	138	198	Kurzawski, 2012	48	19	107	61
	Kang,2009	110	180	261	627	Kang,2009	88	57	224	220
	Ghisdal,2009	146	90	1132	780	09	100	18	771	185
**IGF2BP2(rs4402960)**										
	Yokoyama,2018	7	15	11	43	Yokoyama, 2018	6	5	11	16
	Kurzawski,2012	49	85	109	227	Kurzawski, 2012	41	26	89	79
	Kang,2009	75	215	269	619	Kang,2009	61	84	235	209
	Ghisdal,2009	74	160	615	1297	09	60	57	520	436
**KCNJ11(rs5219)**										
	Yalin,2017	41	75	47	73	Yalin,2017	37	21	43	17
	Dabrowska-Zamojcin,2017	26	44	132	200	- Zamojcin,2017	19	16	103	63
	Yang,2011	123	143	124	216	Yang,2011	88	45	97	73

		Case Group		Control Group		
		Expose	Unexposed	Expose	Unexposed	
**TCF7L2(rs7903146)**	Recessive mode					Homozygote model
	Alagbe,2017	3	11	4	49	7
	Khan,2015	11	31	8	90	Khan,2015
	Yang,2011	5	128	7	163	Yang,2011
	Kurzawski,2011	7	59	10	158	Kurzawski, 2011
	Kang,2009	0	145	0	444	Kang,2009
	Ghisdal,2009	15	103	83	874	09
	Kang,2008b	0	119	0	392	b
**TCF7L2(rs12255372)**						
	Alagbe,2017	1	14	3	55	7
	Yang,2011	7	126	7	163	Yang,2011
	Kurzawski,2011	6	60	11	157	Kurzawski, 2011
**SLC30A8(rs13266634)**						
	Yokoyama,2018	2	9	3	24	Yokoyama, 2018
	Khan,2015	6	36	7	91	Khan,2015
	Kurzawski,2012	33	34	16	152	Kurzawski, 2012
	Kang,2009	18	127	87	357	Kang,2009
	Ghisdal,2009	9	109	83	874	09
	Kang,2008a	22	152	89	361	a
**KCNQ1(rs2237892)**						
	Hwang,2019	25	229	136	712	9
	Dabrowska-Zamojcin,2017	1	34	0	166	- Zamojcin,2017
	Tavira,2011	3	142	2	258	1
	Kang,2009	17	128	66	378	Kang,2009
**PPARγ(rs1801282)**						
	Zhang,2019	0	17	0	112	9
	Kurzawski,2012	0	67	4	164	Kurzawski, 2012
	Wang,2011	0	51	0	72	1
	Yang,2011	2	131	5	165	Yang,2011
	Ghisdal,2009	0	118	8	947	09
**CDKN2A/B(rs10811661)**						
	Yokoyama,2018	2	9	6	21	Yokoyama, 2018
	Kurzawski,2012	53	14	121	47	Kurzawski, 2012
	Kang,2009	53	92	135	309	Kang,2009
	Ghisdal,2009	69	48	630	320	09
**HHEX(rs1111875)**						
	Yokoyama,2018	2	9	7	20	Yokoyama, 2018
	Kurzawski,2012	8	59	31	137	Kurzawski, 2012
	Kang,2009	22	123	37	407	Kang,2009
	Ghisdal,2009	46	72	361	595	09
**IGF2BP2(rs4402960)**						
	Yokoyama,2018	1	10	0	27	Yokoyama, 2018
	Kurzawski,2012	8	59	20	148	Kurzawski, 2012
	Kang,2009	14	131	34	410	Kang,2009
	Ghisdal,2009	14	103	95	861	09
**KCNJ11(rs5219)**						
	Yalin,2017	4	54	4	56	Yalin,2017
	Dabrowska-Zamojcin,2017	7	28	29	137	- Zamojcin,2017
	Yang,2011	35	98	27	143	Yang,2011

	Case Group		Control Group				Case Group		Control Group	
	Expose	Unexposed	Expose	Unexposed			Expose	Unexposed	Expose	Unexposed
**TCF7L2(rs7903146)**					**TCF7L2(rs7903146)**	Heterozygote model				
	3	6	4	27		7	5	6	22	27
	11	13	8	57		Khan,2015	18	13	33	57
	5	88	7	105		Yang,2011	40	88	58	105
	7	32	10	91		Kurzawski, 2011	27	32	67	91
	0	129	0	421		Kang,2009	16	129	23	421
	15	47	83	499		9	56	47	375	499
	0	107	0	375		Kang,2008b	12	107	17	375
**TCF7L2(rs12255372)**					**TCF7L2(rs12255372)**					
	1	7	3	38		7	7	7	17	38
	7	89	7	110		Yang,2011	37	89	53	110
	6	32	11	95		Kurzawski, 2011	28	32	62	95
**SLC30A8(rs13266634)**					**SLC30A8(rs13266634)**					
	2	5	3	11		Yokoyama, 2018	4	5	13	11
	6	16	7	59		Khan,2015	20	16	32	59
	33	4	16	73		Kurzawski, 2012	30	4	79	73
	18	61	87	130		Kang,2009	66	61	227	130
	9	61	83	474		9	48	61	400	474
	22	70	89	137		Kang,2008a	82	70	224	137
**KCNQ1(rs2237892)**					**KCNQ1(rs2237892)**					
	25	128	136	317		9	101	128	395	317
	1	31	0	149		Dabrowska-Zamojcin,2017	3	31	17	149
	3	136	2	237		Tavira,2011	6	136	21	237
	17	76	66	171		Kang,2009	52	76	207	171
**PPARγ(rs1801282)**					**PPARγ(rs1801282)**					
	0	13	0	97		Zhang,2019	4	13	15	97
	0	52	4	125		Kurzawski, 2012	15	52	39	125
	0	45	0	61		Wang,2011	6	45	11	61
	2	103	5	130		Yang,2011	28	103	35	130
	0	92	8	775		9	26	92	172	775
**CDKN2A/B(rs10811661)**					**CDKN2A/B(rs10811661)**					
	2	2	6	4		Yokoyama, 2018	7	2	17	4
	53	0	121	2		Kurzawski, 2012	14	0	45	2
	53	16	135	83		Kang,2009	76	16	226	83
	69	6	630	51		9	42	6	269	51
**HHEX(rs1111875)**					**HHEX(rs1111875)**					
	2	6	7	8		Yokoyama, 2018	3	6	12	8
	8	19	31	61		Kurzawski, 2012	40	19	76	61
	22	57	37	220		Kang,2009	66	57	187	220
	46	18	361	185		9	54	18	410	185
**IGF2BP2(rs4402960)**					**IGF2BP2(rs4402960)**					
	1	5	0	16		Yokoyama, 2018	5	5	11	16
	8	26	20	79		Kurzawski, 2012	33	26	69	79
	14	84	34	209		Kang,2009	47	84	201	209
	14	57	95	436		9	46	57	425	436
**KCNJ11(rs5219)**					**KCNJ11(rs5219)**					
	4	21	4	17		Yalin,2017	33	21	39	17
	7	16	29	63		Dabrowska-Zamojcin,2017	12	16	74	63
	35	45	27	73		Yang,2011	53	45	70	73
